# Valproic acid improves the efficacy of oxaliplatin/fluoropyrimidine-based chemotherapy by targeting cancer stem cell via β-Catenin modulation in colorectal cancer

**DOI:** 10.1038/s41419-025-07902-8

**Published:** 2025-08-01

**Authors:** Maria Serena Roca, Rita Lombardi, Cristina Testa, Federica Iannelli, Laura Grumetti, Tania Moccia, Veronica Barile, Laura Addi, Domenico Memoli, Alessandra Leone, Simone Di Franco, Giorgio Stassi, Antonio Avallone, Francesca Bruzzese, Biagio Pucci, Alfredo Budillon, Elena Di Gennaro

**Affiliations:** 1 Experimental Pharmacology Unit, Istituto Nazionale Tumori Fondazione G. Pascale - IRCCS, Naples, Italy; 2 Experimental Animal Unit -Istituto Nazionale Tumori Fondazione G. Pascale - IRCCS, Naples, Italy; 3 Department of Medicine, Surgery and Dentistry ‘Scuola Medica Salernitana’, University of Salerno, Salerno, Italy; 4 Department of Precision Medicine in Medical, Surgical and Critical Care, University of Palermo, Palermo, Italy; 5 Abdomen Medical Oncology Unit, Istituto Nazionale Tumori-IRCCS- Fondazione G. Pascale, Naples, Italy; 6 Scientific Directorate, Istituto Nazionale Tumori-IRCCS- Fondazione G. Pascale, Naples, Italy

**Keywords:** Cancer therapy, Gastrointestinal cancer

## Abstract

Despite advances in systemic therapeutic approaches, metastatic colorectal cancer (mCRC) patients harboring BRAF or RAS mutations have poor outcomes. Cancer stem cells (CSCs) play central roles in drug resistance and CRC recurrence. Therefore, targeting the epigenetic mechanisms that sustain CSC properties is a promising therapeutic approach. In this study, we report the efficacy of a treatment strategy with the potential to overcome chemotherapy resistance that involves administering the well-known antiepileptic drug and epigenetic agent valproic acid (VPA) and the standard chemotherapy regimen of oxaliplatin/fluoropyrimidine to wild-type CSCs and CSCs with BRAF and RAS mutations in enriched primary spheroid cultures. Notably, we demonstrated that VPA plus chemotherapy was more effective than other epigenetic drug-chemotherapy combinations by inhibiting cell proliferation and clonogenic growth and by inducing apoptosis and DNA damage. Mechanistically, proteomic analysis demonstrated that VPA induced CSC differentiation through the critical target of VPA, β-Catenin. Indeed, VPA promoted the proteasome-dependent degradation of β-Catenin by enhancing its binding to the E2 ubiquitin-conjugating enzyme UBE2a, leading to marked reductions in nuclear and cytoplasmic β-Catenin levels and subsequently decreasing β-Catenin/TCF-LEF target promoter activation. These effects were confirmed in three in vivo CRC xenograft models, including a syngeneic CT26 immunocompetent mouse model, where VPA combined with oxaliplatin/capecitabine chemotherapy and anti-VEGF therapy, a standard first-line treatment for mCRC, significantly suppressed tumor growth and prolonged survival with minimal toxicity. Proteomic analysis of tumor tissues from in vivo CRC models confirmed the VPA-mediated downregulation of CSC markers and β-Catenin.

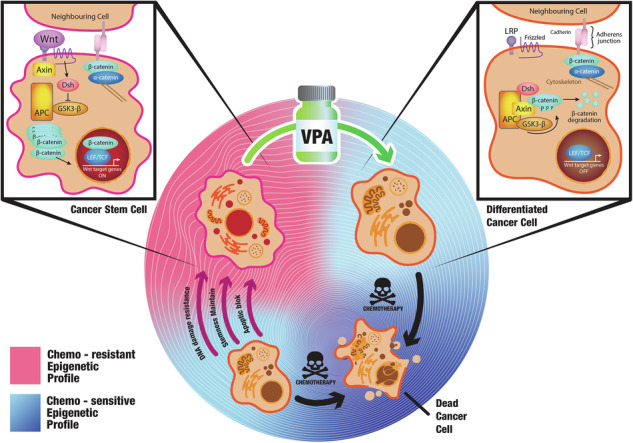

## Introduction

Colorectal cancer (CRC) is a major global health issue. It is the third most commonly diagnosed cancer worldwide and ranks second in terms of mortality [1]. Despite advances in the treatment of metastatic colorectal cancer (mCRC), there remains an unmet need for effective therapies, particularly for patients with BRAF- or RAS-mutated tumors associated with poor prognosis [2]. For these patients, the combination of chemotherapy and anti-VEGF bevacizumab (BEV) is both the only therapeutic option [2, 3]. Therefore, developing innovative combinatorial therapies by repurposing drugs previously approved for non oncological indications might be an attractive strategy to offer more effective treatment options with easy translation in clinical trials [4].

Tumor cells exhibiting stem cell-like characteristics have been detected in various human cancers, including CRC, and are recognized as the primary cause of relapse and metastasis [5]. The intrinsic treatment resistance exhibited by cancer stem cells (CSCs) seems to be a fundamental trait of this subpopulation, and exploiting the differences between CSCs and their more differentiated progeny could be the key to discovering innovative approaches for sensitizing CSCs to chemotherapeutic agents [6]. Cell plasticity, a critical property of CSCs, enables these cells to dynamically transition between stem/nonstem, epithelial/mesenchymal, or quiescent/proliferating phenotypes and switch among these phenotypic states in response to environmental signals [7].

Recent studies in many models of cancer, including CRC, also suggest that in addition to canonical acquired resistance mechanisms, de novo resistant clones can develop due to the emergence of a subpopulation of drug-tolerant persistent (DTP) cells [8, 9]. Interestingly, several characteristics and molecular pathways responsible for DTP resistance to targeted therapy overlap with those responsible for CSC-mediated chemotherapy resistance [10].

The plasticity of cancer cells, including CSCs, is controlled by the dysregulation of genetic and epigenetic factors, including chromatin remodeling, histone modifications, DNA methylation, and regulation by noncoding RNAs [7]. Mutations in epigenetic regulators during the initiation and progression of cancer enhance tumor plasticity, facilitating adaptability and resistance to therapy, thus significantly influencing cell fate [11]. Many mechanisms of epigenetic dysregulation are reversible and heritable, and therapies targeting epigenetic regulation hold promise as effective strategies [11]. In addition, CSC proliferation is tightly regulated by numerous morphogenetic pathways, including the Wnt/β-Catenin, Notch, Hedgehog, TGF-β/BMP, JAK-STAT, and Hippo pathways. Among these pathways, alterations in the Wnt/β-Catenin pathway are critical in the development of CRC and are associated with poor outcome in CRC patient’s [5]. In fact, nearly all CRC patients exhibit either inactivation of the APC gene or activating mutations in β-Catenin. These genetic alterations lead to the stabilization of β-Catenin and continuous activation of the Wnt transcriptional program, even in the absence of extracellular signals [12]. High Wnt activity also confers resistance to various conventional and targeted cancer therapies through multiple mechanisms, including maintenance of the CSC population, enhanced DNA damage repair, increased transcriptional plasticity, and promotion of immune evasion [12]. Additionally, feedback loop upregulation of kinase receptors and activation of the associated pathways have been reported to promote the slow-cycling DTP state, which allows cancer cells to tolerate drug-induced insults, such as AXL-NOTCH3-dependent activation of the β-Catenin signaling pathway [10].

Histone deacetylase inhibitors (HDACis) are a class of epigenetic drugs that are currently used in the clinic or under development as antitumor agents for both solid and hematological malignancies [13, 14]. Our group has investigated HDACis as anticancer agents, both preclinically and clinically, in different cancer models, including CRC [15–19]. Valproic acid (VPA), a widely used and cost-effective generic antiepileptic compound and mood stabilizer, selectively inhibits class I and II HDACs and exhibits anticancer properties [20, 21]. Compared with other HDACis, VPA has a favorable safety profile, indicating its promise for use in combination therapy in cancer patients [18, 19, 21, 22].

In the present study, we report, for the first time, the ability of VPA to induce CSC differentiation, potentiating the effects of chemotherapy in in vitro and in vivo CRC models. This phenomenon is closely associated with the VPA induced dysregulation of the Wnt/β-Catenin pathway. Overall, we suggest a new therapeutic antitumor strategy that increases the efficacy of drugs commonly used in clinical practice for the treatment of patients with CRC.

## Results

### Targeting epigenetic mechanisms sensitizes CSphCs to Oxaliplatin/5′-DFUR treatment

**Fig. 1:**
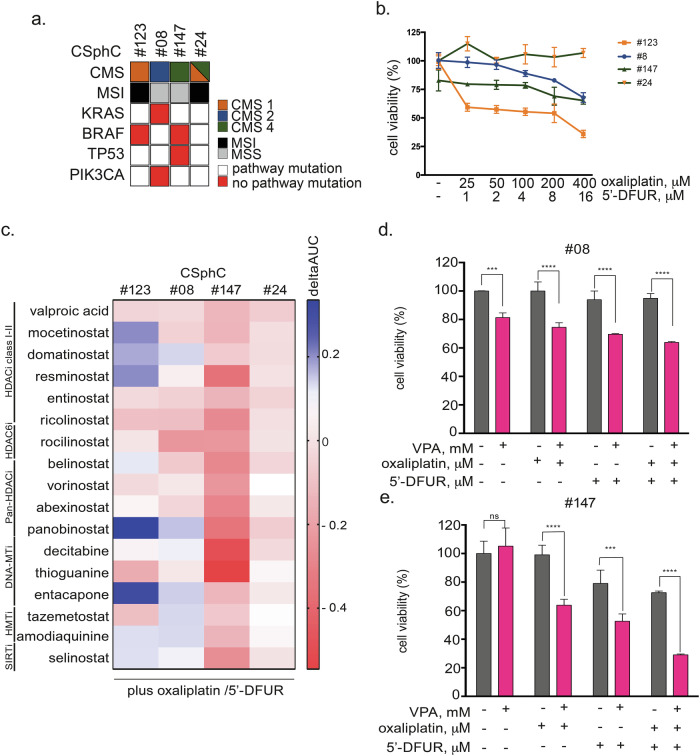
Antiproliferative effects of epigenetic compounds in combination with oxaliplatin/5′-DFUR in CSphCs. **a** CSphC features such as the consensus molecular subtype (CMS), microsatellite instability status (MSI), and mutational status of the CRC driver genes KRAS, BRAF, TP53, and PIK3CA. **b** CSphCs#123, #8, #147, and #24 were treated for 96 h with increasing concentrations of OXA/5′-DFUR, after which cell viability was determined. **c** A small-scale combination drug screen was performed by treating CSphCs with 17 epigenetic compounds at a fixed dose plus the oxaliplatin/5′-DFUR at increasing dosages. The heatmap shows the ΔAUC (AUCepidrug+oxaliplatin/5′-DFUR-AUCoxalipl- atin/5′-DFUR) values for CSphCs#123, #8, #147, and #24; ΔAUC < 0 indicates an increase in the antiproliferative effect of chemotherapy. **d**, **e** CSphCs#8 and #147 were treated for 72 h with VPA (0.5 mM) and/or oxaliplatin (50 μM) and/or 5′-DFUR (1 μM). Cell viability, expressed as a percentage of the control, was assessed by the CellTiter-Glo 3D® Luminescent Assay (see “Materials and Methods”). Significant differences according to a *t*-test are reported (**** indicates *P* < 0.0001, *** indicates *P* < 0.005).

To investigate the potential of using epigenetic modulation to sensitize CR-CSCs to conventional chemotherapy, we developed a small-scale combination drug screening test for CRC cell-derived self-assembled spheroids (CSphCs), a model characterized by clear CSC enrichment [23]. We evaluated 17 epigenetic compounds at fixed doses, selected from the literature, plus OXA and the capecitabine metabolite 5′-DFUR at increasing doses (Supplementary Fig. 1a) in four primary CSphCs, #08, #24, #123, and #147. Different driver gene mutations (KRAS, BRAF, TP53, and PIK3CA), mismatch repair status (MSI or MSS) and consensus molecular subtypes (CMSs) characterize these CSphCs (Fig. 1a). Moreover, this CSphC panel showed variable sensitivity to OXA/5′-DFUR (Fig. 1b) and the epigenetic compounds (Supplementary Fig. 1b). Among the epigenetic drugs evaluated, the HDACis demonstrated the highest efficacy in increasing the sensitivity of all CSphCs to OXA/5′-DFUR treatment, as determined by the ΔAUC values (Fig. 1c).

Interestingly, VPA alone exhibited similar antiproliferative effects in all four CSphCs (Supplementary Fig. 1b). Notably, VPA was tested at 0.5 mM, a low dose, easily achievable in the plasma of epileptic patients treated with a standard regimen [24]. The potentiation of the antitumor effect of VPA was particularly notable in the more aggressive and chemoresistant KRAS-mutant (CSphC #8) and BRAF-mutant (CSphC #123 and CSphC #147) spheroids (Fig. 1d, e and Supplementary Fig. 2).

Taken together, these findings suggest that cotreatment with an HDACi, including VPA, increases the susceptibility of CSCs to the antitumor activity of conventional chemotherapeutic agents.

### VPA affects the viability and phenotype of colon CSCs

**Fig. 2:**
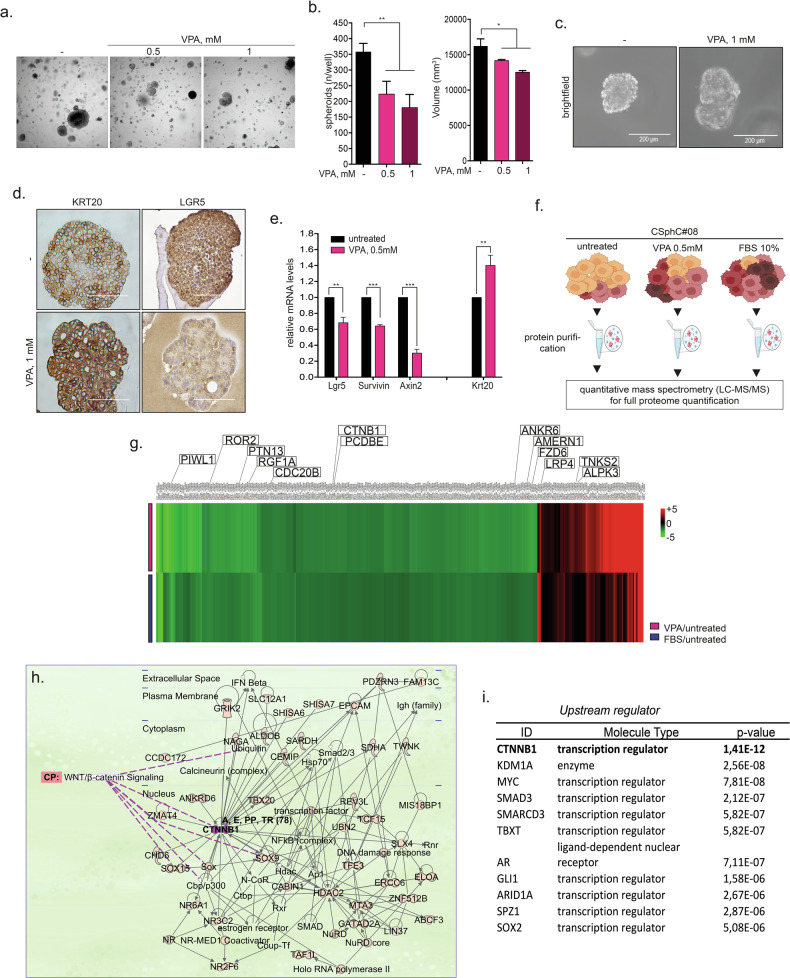
VPA affects innate stem cell properties and induces a differentiation-like phenotype. **a**, **b** A total of 4000 CSphCs#08 per ml were plated in Matrigel (70%), and after 24 h, VPA (0.5 and 1 mM) was added for 24 h. Next, 72 photographs were captured of each well, and quantitative and qualitative analyses were performed with Opera Phoenix HTS and Harmony software. **c** Phase‒contrast analysis of CSphC#8 grown in Matrigel and treated as indicated for 2 days. One representative of four independent experiments carried out with CSphC#8 is shown. Scale bars, 200 μm. **d** Immunohistochemical analysis of CK20 and Lgr5 in CSphC#8 growing in Matrigel for 24 h and treated for 16 h with 0.5 mM VPA. Scale bar, 200 μm. **e** Evaluation of the mRNA expression of the differentiation marker cytokeratin 20 (CK20) and stem cell markers (Lgr5, Survivin, and Axin2) upon VPA treatment (0.5 mM, 16 h). **f** Full proteome identification via LC‒MS/MS technology was performed as schematically reported. **g** Heatmap showing the 515 proteins with similar changes in expression level in CSphC#08 upon VPA treatment under differentiation-induced conditions (10% FBS) compared with that in untreated CSphC#08. The criteria were a *P* value less than 0.05 and a fold change of less than +1 or −1 in one experimental group. The colorimetric scale is fixed at +5 and −5. The 15 boxes highlighted in the heatmap represent previously established stem cell-associated genes. **h** Gene–gene network generated by IPA starting from the 515 differentially expressed proteins. The central hierarchical dominant hub in the network was β-Catenin, and the main canonical pathway was Wnt/β-Catenin signaling. **i** The top ten upstream transcription regulators of the 515-gene set are reported, with β-Catenin as the most significant regulator. The Ingenuity® Pathway Analysis network legend can be accessed at http://qiagen.force.com/KnowledgeBase/articles/ Basic_Technical_Q_A/Legend. Significant differences according to a t-test are reported (*** indicates *P* < 0.0001, ** indicates *P* < 0.001, * indicates *P* < 0.05).

Along with other HDACis, VPA induces several pleiotropic effects in cancer cells that modulate cellular homeostasis, including cell differentiation [25, 26]. Accordingly, we observed that in CSphC #08, VPA significantly reduced the size and number of spheroids (Fig. 2a, b) and induced marked morphological changes (Fig. 2c–e). Specifically, distinct changes in polarization were observed in the majority of the relapsed structures upon VPA treatment (Fig. 2c), paralleled to the increased expression of the differentiation marker cytokeratin 20 (krt20) and a reduction in the expression of stem cell markers Lrg5, Survivin and Axin2 at both the protein (Fig. 2d) and mRNA levels (Fig. 2e). These effects were confirmed in CSphCs #123 and #147 (Supplementary Fig. 3a–d). Next, to demonstrate the selective differentiation-inducing effect of VPA in tumor cells, we evaluated the expression of the CSC markers Sox9, Axin2, Cd44, and Ascl2 in organoids isolated from healthy mouse intestines. Notably, we observed that VPA upregulated the expression of the analyzed stemness markers at the transcriptional level (Supplementary Fig. 4), which is in accordance with previous reports [27], indicating that VPA has the opposite effect in tumor cells compared with that in healthy organoids.

To further explore the mechanisms underlying the effects of VPA, we conducted whole-proteome expression profile analysis on CSphC #08 untreated, treated with VPA (24 h), or cultured under differentiation-inducing conditions (10% fetal bovine serum [FBS] for 24 h) (Fig. 2f).

We identified 589 differentially expressed proteins in the VPA- and 10% FBS-treated CSphC #08 spheroids compared with the untreated CSphC #08 spheroids (*P* value ≤ 0.05). As shown in Fig. 2g, we identified 515 proteins with a fold change in expression that was ±1.5-fold that of the control (considered 0) in at least one of the two treatment groups. Specifically, we identified proteins whose expression changed in the same direction (403 downregulated and 112 upregulated) in both VPA- and 10% FBS-treated spheroids (Fig. 2g). Among the identified proteins modulated by VPA, some have been previously established as intestinal stem cell-associated proteins [23] (Fig. 2g and Supplementary Fig. 5).

Moreover, to identify the regulatory networks in which the identified proteins are involved and thus to elucidate their biological functions and relationships, we used Ingenuity Pathway Analysis (IPA) software. Interestingly, the analysis revealed that in addition to those associated with cancer and gastrointestinal disease, several of the top functions associated with the identified protein interaction networks, such as cell growth and proliferation, cell death and survival, DNA replication, recombination and repair, and the cellular response to therapeutics, are involved in the induction of chemoresistance (Table 1).

**Table 1:** Disease and functions.

Category	*p*-value
Cancer	4.72e-21–4.92e-03
Gastrointestinal disease	7.99e-16–4.80e-03
Cellular assembly and organization	1.26e-07–4.98e-03
Cellular function and maintenance	1.26e-07–4.98e-03
Tissue development	1.32e-07–5.11e-03
Cell morphology	2.02e-06–4.98e-03
Cellular development	6.05e-06–5.11e-03
Cellular growth and proliferation	6.05e-06–5.11e-03
Cell death and survival	1.28e-05–4.66e-03
Cell cycle	2.62e-05–5.11e-03
OrganismaL Survival	3.48e-05–4.98e-04
Cell-to-cell signaling and interaction	9.85e-05–3.59e-03
Cellular movement	9.85e-05–4.73e-03
DNA replication, recombination, and repair	1.69e-04–4.98e-03
Post-translational modification	1.60e-03–4.10e-03
Cellular response to therapeutics	2.01e-03–2.65e-03

Furthermore, the top network among the 10 identified by IPA was WNT/*β*-Catenin signaling, which was composed of 40 clustered proteins with *β*-Catenin as a relevant central hub (Fig. 2h). Interestingly, we also found that β-Catenin was the most significant upstream transcriptional regulator within this network (*p*-value = 1.41 × 10^−12^), followed by other established HDACi targets [5] (Fig. 2i).

In summary, we showed that chemotherapy potentiation in CSCs driven by VPA occurs in parallel with the induction of differentiation, and this effect might occur via modulation of the WNT/*β-*Catenin signaling pathway.

### VPA modulates the WNT/β-Catenin signaling pathway

**Fig. 3:**
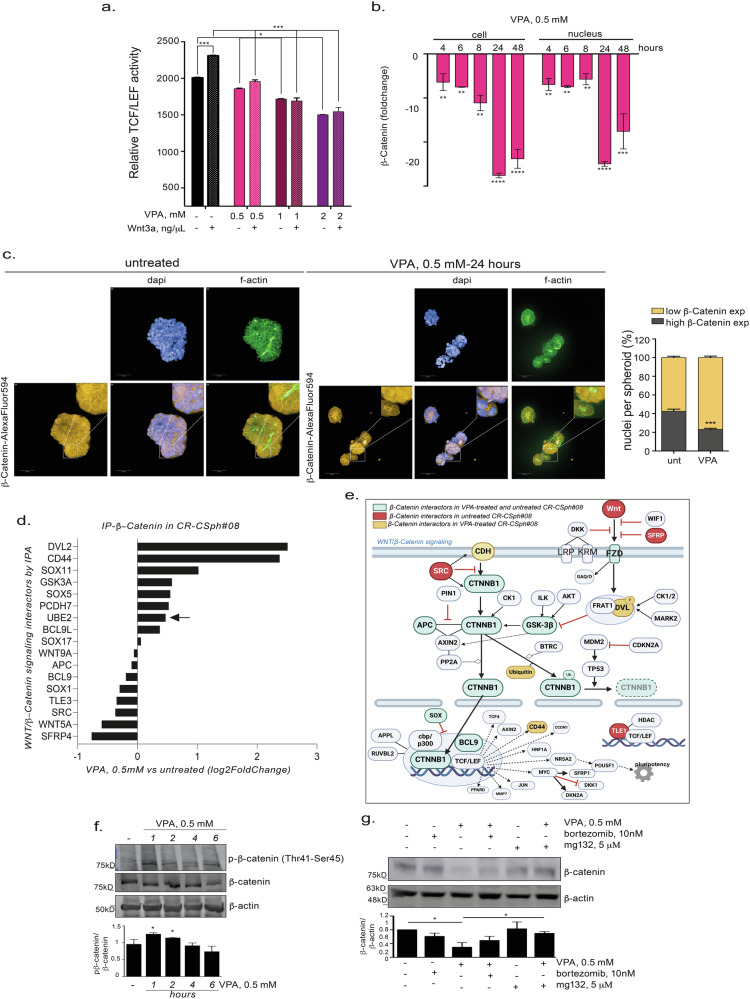
VPA modulates the Wnt signaling pathway and β-catenin expression. **a** CSphC#147 were treated with VPA (0.5, 1, or 2 mM) alone or with 25 ng/μl wnt3a for 24 h, and TCF/LEF luciferase reporter activity was evaluated with a dual luciferase kit. The error bars represent the standard errors of the mean (SEMs) (*n* = 4); the reported data are representative of three independent experiments. **b** CSphC #147 was treated with VPA (0.5 mM) for the indicated durations, after which the cells were fixed and stained with βCatenin-Alexa 594. Cellular and nuclear β-Catenin protein expression was measured with Harmony software. **c** Representative overview confocal images displaying β-Catenin expression, F-actin fibers (stained with phalloidin-FITC) and nuclei (stained with DAPI) in CSphC#08 that were untreated or treated with 0.5 mM VPA; magnification, 40×. Scale bar: 50 μm and inset magnification 80×. β-Catenin nuclear intensity quantified by Harmony software for each cell in each spheroid and then clustered into β-Catenin low-expression nuclei and β-Catenin high-expression nuclei considering the average β-Catenin intensity in each spheroid. The error bars represent the SEMs (spheroids *n* = 30); the reported data are representative of three independent experiments. **d** Proteins involved in WNT/β-Catenin signaling identified via label-free MS/MS upon IP of β-Catenin starting from 500 ng of total protein lysate and identified by IPA software (Mascot score > 50, at least one unique peptide with a *P* value < 0.05). The fold change of mascot score represents the presence of proteins in VPA-treated versus untreated CSphC#08. **e** A representative WNT/β-Catenin signaling pathway where the β-Catenin interactors reported in the protein list in d are shown in green, red, and orange. **f** β-Catenin phosphorylation and expression in CSphC#08 treated with VPA (0.5 mM) and collected after 1, 2, 4, and 6 h. β-Actin served as a loading control. The graph at the bottom shows the expression of phospho-β-Catenin normalized to that of β-Catenin and β-actin. **g** β-Catenin protein expression in CSphC#08 treated with VPA (0.5 mM) and/or bortezomib (10 mM) and/or mg132 (5 μM) for 6 h. β-actin served as a loading control. The graph at the top shows the fold change in expression compared with that in the untreated sample. Significant differences according to a *t*-test are reported (*** indicates *P* < 0.0001, ** indicates *P* < 0.001, * indicates *P* < 0.05).

Based on the data reported above, we delved deeply into the effects of VPA on the Wnt pathway and β-Catenin signaling cascade. First, we evaluated the effect of VPA on TCF/LEF, the main promoter triggered by Wnt pathway activation and β-Catenin translocation to the nucleus in CSphC#08 cells and demonstrated a dose-dependent reduction in promoter activity within 24 h (Fig. 3a). Notably, the effect of VPA on TCF/LEF promoter activity remained consistent even when VPA was administered simultaneously with Wnt3a (25 ng/µL), a frizzled receptor family member ligand and an activator of TCF/LEF promoters (Fig. 3a). Moreover, this decrease in activity was associated with time-dependent reductions in the nuclear and cytoplasmic levels of β-Catenin induced by VPA in adherent CSphC#08 cells, as shown by β-Catenin immunofluorescence staining and subsequent quantification (Fig. 3b and Supplementary Fig. 6b).

Considering the complex structures of spheroids, which are composed of many cells with different states of Wnt pathway activation, we then examined the percentage of cells within each spheroid displaying elevated nuclear β-Catenin levels and the ability of VPA to decrease this proportion via immunofluorescence staining. Among the CSphC#08 cells, 42.55 ± 2.3% had nuclear β-Catenin levels 10 times greater than the average intensity measured in all the cells within each spheroid. After 24 h of treatment with 0.5 mM VPA, the percentage of cells with high nuclear β-Catenin levels decreased by half to 23.15 ± 1.2% (Fig. 3c). The ability of VPA to reduce the population of cells characterized by high levels of β-Catenin within the nucleus was further confirmed in CSphC#147, which inherently presented fewer nuclei expressing high levels of β-Catenin (13.37 ± 1.5%) compared to CSphC#08 (Supplementary Fig. 6). Upon VPA treatment, the percentage of cells with high nuclear β-Catenin expression decreased to 3.16 ± 1.4% (Supplementary Fig. 6).

Next, to elucidate the mechanism by which VPA modulates β-Catenin, we performed immunoprecipitation of the β-Catenin protein in untreated CSphC#08 and those treated with 0.5 mM VPA for 24 h, and LC-MS/MS technology was applied to identify the βCcatenin-interactome.

Through Mascot search engine software, we identified 884 and 1023 proteins in untreated and VPA-treated cells, respectively, which showed significant binding to β-Catenin (with a Mascot score >50; *p* ≤ 0.05). Using IPA software, we identified the proteins reported in the literature to be directly related to β-Catenin, and 188 proteins were selected from untreated CSphC#08 and 245 from VPA-treated CSphC#08 (see the raw data link). We focused on the proteins identified by IPA as linked to “WNT/β-Catenin signaling” (Fig. 3d, e) and found four β-Catenin interactors present in both the untreated and VPA-treated spheroids: GSK3, APC, BCL9, and SOX (highlighted in green in Fig. 3d, e). Conversely, we also identified four interactors specific to either the untreated cells (SFRP, SRC, TLE, and WNT; highlighted in red in Fig. 3d, e) or VPA-treated spheroids (CD44, DVL, CDH, and ubiquitin; highlighted in orange in Fig. 3d, e). Notably, we observed that VPA increased the interaction between β-Catenin and UBE2A (UniprotKB entry names), the ubiquitin-conjugating enzyme E2A responsible for the proteasome-dependent degradation of β-Catenin.

Considering that β-Catenin protein levels are regulated primarily by proteasome-dependent degradation, mainly through negative phosphorylation at threonine 41 and serine 45 by GSK3β [5], we investigated whether the observed β-Catenin downregulation could be attributed to this mechanism.

Consistent with this hypothesis, VPA (0.5 mM) treatment induced an increase in β-Catenin phosphorylation at threonine 41 and serine 45 in CSphC#08 within 1 h, which could be sustained for up to 6 h (Fig. 3f). Notably, GSK3β protein expression was not affected by VPA (Supplementary Fig. 7), and this protein was found to interact with β-Catenin in both untreated and VPA-treated spheroids (Fig. 3d, e), suggesting alternative functional regulation mechanisms. Moreover, 6 h of cotreatment with bortezomib or mg132, two proteasome inhibitors, reversed the VPA-mediated reduction in β-Catenin protein expression (Fig. 3g).

Taken together, these results suggest that VPA downregulates β-Catenin and decreases its function by modulating the binding with its interactors and by promoting its proteasome-dependent degradation by inducing threonine 41 and serine 45 phosphorylation.

### VPA potentiates the antiproliferative, proapoptotic and DNA damage effects of Oxaliplatin/5′-DFUR treatment

**Fig. 4:**
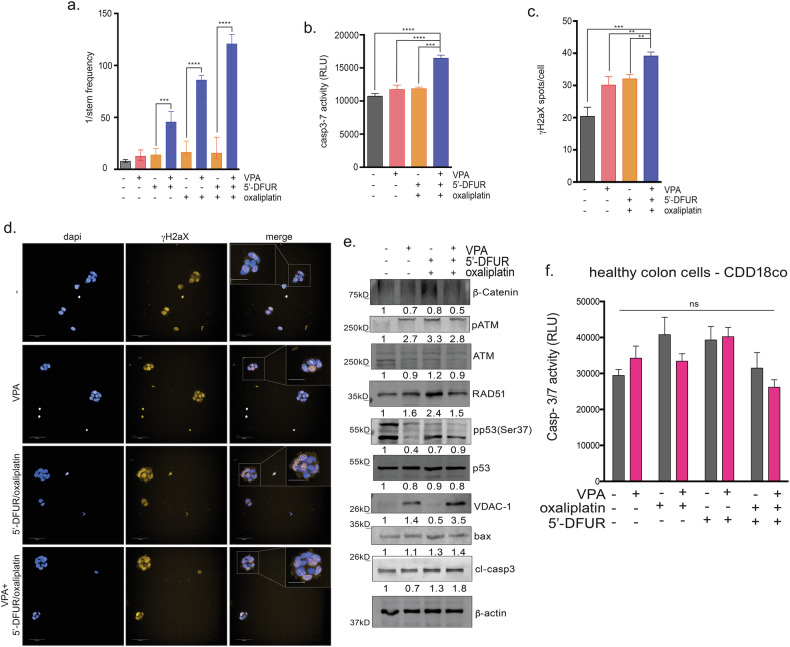
VPA potentiates the antitumor effects of oxaliplatin/5′-DFUR. CSphCs were treated with 0.5 mM VPA, 100 nM oxaliplatin, and 4 μM 5′-DFUR. **a** Limiting dilution assay performed on CSphC#08 without or with 24 h of treatment with VPA plus oxaliplatin and 5′-DFUR plated in ultralow attachment 96-well plates without additional treatment for three weeks. Clonal frequency was evaluated with the extreme limiting dilution analysis limdil function as described in the “Materials and Methods” section. **b** Apoptosis was evaluated by a Caspase 3/7 activity assay in CSphC #08 treated as previously described. **c**, **d** DNA damage was analyzed in CSphC #08 by visualizing foci of the double-strand break marker γH2AX. CSphCs were treated for 24 h with or without VPA and oxaliplatin/5′-DFUR. CSphCs were stained for γH2AX (orange), and DAPI was used to stain the nuclei (blue) before Opera Phenix HTS microscopy measurements. The number of spots for each cell was analyzed by Harmony software. Representative images showing γH2AX-positive nuclear foci at 40× magnification and insert magnification 80×. Significant differences according to the *t*-test are reported (*** indicates *P* < 0.0001, ** indicates *P* < 0.001, * indicates *P* < 0.05). **e** β-Catenin, pATM, ATM, pp53(ser37), p53, RAD51, BAX, VDAC-1, and GPX4 protein expression and cleaved caspase 3 levels in CSphC #147 treated with VPA alone or in combination with chemotherapeutics for 24 h. β-Actin served as a loading control. The data shown are representative of three independent experiments. **f** The healthy colon cell CDD18co were treated for 72 h with VPA (0.5 mM) and/or oxaliplatin (50 μM) and/or 5′-DFUR (1 μM). Cell viability, expressed as a percentage of the control, was assessed by the CellTiter-Glo 3D® Luminescent Assay (see “Materials and Methods”). Significant differences according to the t-test are reported (ns indicates *P* > 0.05).

Next, we evaluated how VPA affects the mechanisms that contribute to the intrinsic resistance of CSCs to chemotherapy, such as their clonogenic capacity, ability to block apoptosis induction, resistance to DNA damage and inability to die upon the accumulation of genetic lesions [28, 29]. We first showed, via a limiting dilution assay, that the clonogenic capacities of CSphCs#08, #147 and #123 were significantly reduced when the cells were treated with chemotherapeutics in combination with VPA for 24 h (Fig. 4a and Supplementary Fig. 8a). We also demonstrated that the balance between CSC apoptosis and DNA damage could be modulated by VPA via a dose-dependent reduction in the proapoptotic factor Bcl-2 and an increase in the DNA damage marker γH2ax at the protein level (Supplementary Fig. 8b). Accordingly, we demonstrated that, compared with monotherapy, VPA induced a significant increase in apoptosis after 48 h of cotreatment with OXA/5′-DFUR, as determined by caspase 3/7 cleavage or the number of annexin-V-positive cells (Fig. 4b and Supplementary Fig. 8c). In line, the number of γH2ax foci in CSphCs treated with VPA alone or in combination with chemotherapy was higher in the combination treatment group compared to single drug treatment (Fig. 4c, d and Supplementary Fig. 8d).

Interestingly, the induction of apoptosis, reported as caspase 3 cleavage, was coupled with a reduction in β-Catenin protein expression in CSphC#147 treated with the triple combination of VPA and both chemotherapeutics for 24 h (Fig. 4e). In agreement with our previously published results [17], the combination of VPA, 5′-DFUR, and oxaliplatin induced DNA damage and apoptosis via ATM and mitochondrial pathway, as shown by increased phosphorylation of ATM, as well as modulation of RAD51, VDAC-1 and BAX expression (Fig. 4e and Supplementary Fig. 8e). Notably, we treated the normal colon epithelial cell line CCD18Co with VPA alone and in combination with oxaliplatin and 5′-DFUR for 48 h, evaluating caspase 3/7 activity. Our findings indicated that VPA did not exhibit any synergistic effects plus chemotherapy, suggesting that the combination treatment selectively targets cancer cells (Fig. 4f).

Taken together, our findings suggest that the innate CSC properties of CR-CSCs are compromised by VPA, thus favoring the cytotoxic effects of the chemotherapeutic agents.

### VPA sensitizes colon CSCs to chemotherapy in vivo

**Fig. 5:**
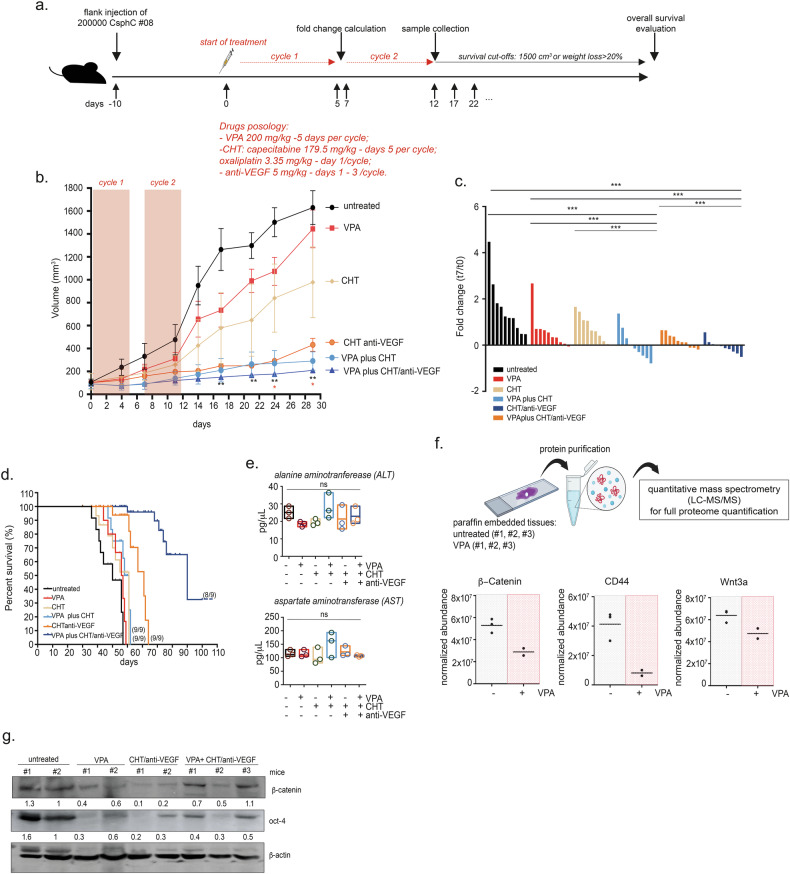
In vivo synergistic antitumor effect of VPA plus CHT. **a** Schematic of the in vivo xenograft experiment, including timeline and agent concentration. CSphC#08 was s.c. injected into athymic mice as described in the “Materials and Methods”. When the established tumors were palpable, the mice were treated as reported in the figure. The mice were sacrificed when the tumor volume became greater than 1500 mm^3^ and/or when a 20% reduction in weight was observed. **b** CSphC#08 tumor volume curves. The means ± SEMs are reported for each single or combination treatment at each time point. The statistically significant results are shown in black for the comparison of the untreated, VPA, CHT, and VPA + CHT groups and in red for the comparison of the untreated, VPA, CHT/anti-VEGF, and VPA + CHT/anti-VEGF groups. **c** A synergistic effect was observed after 1 treatment cycle, as shown by the waterfall plot of the fold change in tumor volume compared with the baseline (day 0, start of treatment) in the transplanted tumors. *P* values were calculated via a two-tailed, unpaired Student’s *t*-test. (*** indicates *P* < 0.0001, ** indicates *P* < 0.001, * indicates *P* < 0.05). **d** Kaplan–Meier curves comparing survival in the single and combination treatment groups. The number of mice per treatment condition is indicated. **e** Serum alanine aminotransferase (ALT) and aspartate aminotransferase (AST) levels were evaluated in 3 mice from each group at the end of two cycles of treatment. **f** Full proteome identification by LC‒MS/MS, starting from a 1 mm^2^ spot obtained from embedded untreated (*n* = 3) and VPA-treated (*n* = 2) tumor samples, as schematically noted. The normalized abundances of β-Catenin, CD44, and wnt3a are reported. **g** β-Catenin and oct-4 protein expression in CSphC #08 tumor tissues collected at the end of treatment. β-Actin served as a loading control.

To confirm the therapeutic potential of the combination of VPA and commonly used chemotherapeutic agents for the treatment of mCRC in vivo, tumor xenografts were generated by subcutaneously injecting CSphC#08 and #147 into nude mice. The mice were subsequently treated with vehicle alone, VPA, or suboptimal doses of OXA plus capecitabine (CHT), with or without an anti-VEGF antibody, an established drug for the treatment of CRC patients with RAS or BRAF mutations, or with VPA plus CHT with or without anti-VEGF, as illustrated schematically in Fig. 5a and Supplementary Fig. 9a.

The addition of VPA to CHT or CHT plus the anti-VEGF antibody significantly inhibited tumor growth and clearly potentiated the efficacy of CHT or CHT plus anti-VEGF antibody treatment (Fig. 5b and Supplementary Fig. 9b). We observed that the tumor burden, measured for each individual mouse, was significantly lower in the VPA plus CHT or VPA plus CHT/anti-VEGF groups after one treatment cycle than in the other treatment groups (Fig. 5c and Supplementary Fig. 9c). Notably, the synergistic effect of VPA plus CHT and of VPA plus the CHT/anti-VEGF antibody resulted in a significant increase in mouse survival (Fig. 5d and Supplementary Fig. 9d). Additionally, the combined treatments were well tolerated, with no significant alterations in liver enzyme (aspartate transaminase (AST) and alanine aminotransferase (ALT) levels (Fig. 5e and Supplementary Fig. 9e).

Next, to gain insight into the mechanism in vivo, we performed a proteomic analysis of three CSphC#08 tumor tissue samples collected from untreated and VPA-treated mice at the end of treatment via LC-MS/MS technology. Using Progenesis QI for proteomics, 112 proteins were quantified using the following criteria: fold change ≥ 2 and ANOVA test *P* value ≤ 0.05 (link to raw data). Among these proteins, 34 were upregulated and 75 were downregulated upon VPA treatment. Pathway enrichment analysis of the 112 differentially expressed proteins carried out with IPA software revealed five networks (link to raw data), and the most enriched network was centered on β-Catenin and included 21 proteins (Supplementary Fig. 10a). Interestingly, consistent with the in vitro data, we demonstrated a reduction in the protein levels of β-Catenin, CD44, and Wnt3a, the canonical Wnt signaling activator, in the VPA-treated group compared with those in the untreated group (Fig. 5f). Accordingly, we also found that VPA, either alone or in combination with CHT, can decrease the protein expression of β-Catenin in tumor tissue samples from mice with CSphC#08 and #147 xenografts (Fig. 5g and Supplementary Fig. 9f) and of oct-4 in tumor tissue samples from mice with CSphC#08 (Fig. 5g). These effects were paralleled by a substantial increase of γH2ax protein levels and of PARP cleavage in the group receiving the triple combination treatment, suggesting that both DNA damage and apoptosis were induced (Supplementary Fig. 9g) in CSphC#147. These data were not fully confirmed in CSphC#08, as the tumors in the control and VPA-treated groups had already reached considerable sizes, thereby activating apoptotic and damage mechanisms, probably due to hypoxia (Supplementary Fig. 10b). Finally, we confirmed the synergistic antitumor effect reported above using an in vivo syngeneic immunocompetent mouse model. Specifically, tumor xenografts were generated by subcutaneously injecting the RAS-mutant CRC mouse cell line CT26 into Balb/c mice, which were then treated with vehicle alone, VPA, suboptimal doses of CHT with an anti-VEGF antibody, or VPA plus CHT and anti-VEGF, as illustrated in Supplementary Fig. 10a. The addition of VPA to CHT plus anti-VEGF antibody significantly inhibited tumor growth and clearly potentiated the treatment efficacy of CHT plus anti-VEGF (Supplementary Fig. 11b). We also observed that the tumor burden, measured for each mouse, was significantly lower in the VPA plus CHT/anti-VEGF group than in the other treatment groups after one treatment cycle (Supplementary Fig. 11c).

Taken together, these data demonstrate that the addition of VPA to standard chemotherapy plus anti-VEGF treatment provided a significant antitumor benefit in different RAS/BRAF-mutated CRC in vivo models.

Notably, these data also validated the mechanistic findings observed in vitro regarding the ability of VPA to target the stem-like resistant phenotype of CSCs and their associated cell traits, including apoptosis and DNA damage, in vivo.

## Discussion

Antitumor strategies after metastasis are successful if they can induce regression and/or prevent disease progression, thereby improving patient survival. Notably, the treatment landscape for RAS- or BRAF-mutated mCRC is evolving, with emerging targeted therapies such as RAS inhibitors and the recently published Breakwater trial on BRAF targeting [30].

However, chemotherapy, which is still the backbone of all standard first-line approaches to treat mCRC, often has limitations such as poor selectivity, high systemic toxicity, and drug resistance development, which significantly limit its clinical effectiveness.

In this study, we identified the antiepileptic VPA, a widely used HDACi, as the best agent to potentiate dual-OXA/fluoropyrimidine chemotherapy efficacy and overcome chemotherapy resistance in wild-type and BRAF- and RAS-mutated CSC-enriched primary spheroid CRC cultures via low-throughput epigenetic drug screening. Specifically, we demonstrated that VPA combined with chemotherapy had a significant antiproliferative effect, reducing clonogenic growth and inducing apoptosis and DNA damage more effectively than did chemotherapy alone. Notably, VPA was administered at dosages that are easily reached in the plasma of patients undergoing treatment for its original antiepileptic indications [31]. Importantly, we also reported evidence suggesting for the first time that VPA potentiates chemotherapy by facilitating the selective differentiation of CSphCs. Conversely, we showed that VPA induced the expression of stem cell markers in organoids isolated from healthy mouse intestines, which is in line with previous reports indicating that VPA may contribute to maintaining the self-renewal ability of intestinal stem cells [27, 32]. This latter observation might also suggest that VPA, while potentiating the efficacy of chemotherapy against cancer cells, should not enhance, but rather reduce, side effects. Our findings align with the evidence reported by our group and others, demonstrating that HDACis, when in combination with chemotherapeutics, can increase apoptosis, induce cell cycle arrest, and reverse drug resistance in various types of cancer. Specifically, we previously demonstrated a consistent synergistic mechanistic interaction between HDACis and fluoropyrimidines on the basis of the specific modulation of critical enzymes involved in fluoropyrimidine metabolism, such as thymidylate synthase and thymidine phosphorylase, in colorectal [16, 17], breast [33], and head and neck cancers [34]. Interestingly, we have also shown the synergistic antitumor interactions of HDACis plus fluoropyrimidines with DNA damage approaches such as radiotherapy [17] or cisplatin administration [34]. Moreover, Zhijun et al. reported that low concentrations of the HDACi domatinostat sensitized CRC cells to the effects of OXA [35]. Several studies have also shown that HDACis, such as VPA, can preferentially target the reversible, nongenetic features of CSCs [5]. In this context, our group previously demonstrated that the induction of mitochondrial and cellular oxidative stress, along with the modulation of the transcription factor FOXM1 upon treatment with the HDACi domatinostat, sensitized the CSC subpopulation in pancreatic cancer models to chemotherapeutics [36]. Moreover, we have shown that VPA plus the cholesterol-lowering drug simvastatin synergistically sensitizes prostate cancer cells to docetaxel through CSC targeting via the mevalonate pathway/YAP axis modulation [37, 38].

Notably, our study highlighted β-Catenin as a critical target of VPA in colon-CSC-enriched models. Specifically, we demonstrated that VPA promotes β-Catenin degradation in CRC CSphCs through proteasome-dependent mechanisms, leading to a marked reduction in β-Catenin levels in both the cytoplasm and nucleus, subsequently diminishing the activation of β-Catenin/TCF/LEF target promoters. Additionally, VPA enhanced the binding of β-Catenin to UBE2a, the E2 ubiquitin-conjugating enzyme responsible for its degradation. Interestingly, Wnt/β-catenin, along with other morphogenetic pathways, is frequently dysregulated in cancer via epigenetic mechanisms [39]. Moreover, inhibiting Wnt signaling through bone morphogenetic protein (BMP) signaling [40] or inhibiting the Notch pathway [41] has been shown to induce the differentiation of colon CSCs, thereby increasing their sensitivity to conventional chemotherapy. Although many interventions targeting Wnt/β-catenin signaling have shown promise in clinical trials of gastrointestinal cancers, no drugs have been approved to effectively target this pathway [3, 42]. Our findings are consistent with those of Ozman Z et al., who reported that VPA increased Snail expression and induced epithelial–mesenchymal transition (EMT) in breast cancer cells by downregulating E-cadherin, GSK3β, AKT, and β-Catenin [43]. Similarly, additional previous evidence suggests that HDAC inhibition results in decreased β-catenin nuclear localization, leading to a significant inhibition of cell proliferation [44, 45]. Notably, computational approaches such as BENEIN have demonstrated the potential of perturbing HDAC2 as master regulator to drive differentiation and revert cancer cells to normal-like states, reinforcing the significance of our findings on VPA’s role as an HDAC inhibitor [46]. However, further studies are required to determine the exact components of the Wnt/β-catenin pathway that are modulated by VPA. In addition, we cannot exclude the possibility that additional mechanisms are involved, considering that multiple pathways are targeted by VPA, as also shown by our proteomic analysis and the top upstream regulators that emerged.

Finally, we provide proof-of-concept in vivo studies in three different xenograft mouse models, including a reliable immunocompetent syngeneic mouse model, which demonstrated the feasibility and benefit of combining VPA with chemotherapeutics employed for the standard first-line treatment for mCRC patients. Specifically, we demonstrated for the first time the significant positive outcomes of VPA combined with CHT and an anti-VEGF antibody in BRAF- and KRAS-mutant CRC models with a poor outcome. Consistent with our results, a previous study by Pili R and colleagues revealed that combining the HDACi vorinostat with BEV (anti-VEGF) was both safe and more effective than BEV alone in renal cancer models [47]. Furthermore, using broad-spectrum LC‒MS/MS proteomic technology, we validated the molecular mechanisms observed in vitro, confirming that both CSC markers and β-catenin were downregulated in tumor tissues derived from VPA-treated mice compared with those derived from untreated control mice.

In summary, our in vitro and in vivo results collectively suggest that repurposing a safe and generic drug, such as VPA, as an anticancer agent could significantly potentiate the standard treatment of patients with mCRC, particularly those with challenging RAS- and BRAF-mutated tumors. Nevertheless, further studies will be required to investigate the potential effects of VPA in combination with emerging BRAF inhibitors to better define its role in the evolving treatment landscape of BRAF-mutant mCRC.

On these bases, we recently launched an ongoing randomized phase 2 clinical study evaluating VPA in combination with BEV and OXA/fluoropyrimidine regimens in mCRC patients harboring RAS mutations (REVOLUTION trial, NCT01898104) [31]. Exploratory correlative biomarker studies on the samples collected from patients enrolled in the trial will eventually confirm our mechanistic hypothesis and lead to the identification of patient subsets that would benefit the most from the association of VPA with standard therapy.

## Materials and methods

### CRC spheroid cell (CSphC) culture

Colorectal-CSphCs (CSphCs) #08 and #24 were kindly provided by the Stassi G Laboratory [48]. CSphCs #123 and #147 were kindly provided by the Medema JP Laboratory [49]. The CSphCs were cultured in advanced DMEM/F12 (Thermo Fisher Scientific) containing N2 supplement (Thermo Fisher Scientific), B27 supplement (Thermo Fisher Scientific), 2 mM L-glutamine (Lonza), 10 mM HEPES (Thermo Fisher Scientific), 1 mM N-acetyl cysteine (Sigma‒Aldrich), 10 mM nicotinamide (Sigma‒Aldrich), 50 ng/ml epidermal growth factor (Thermo Fisher Scientific), 50 ng/ml basic fibroblast growth factor (Thermo Fisher Scientific) and penicillin (100 IU/ml)–streptomycin (100 μg/ml). The healthy human colon cancer cell lines CCD-18Co (CRL-1459) were purchased from the American Type Culture Collection. The cell line was cultured in Eagle’s Minimum Essential Medium (Thermo Fisher Scientific) with the addition of FBS to a final concentration of 10% and penicillin (100 IU/ml)–streptomycin (100 μg/ml). The cultures were maintained as spheroids in ultralow adherent flasks (Corning) at 37 °C and 5% CO_2_, and confirmed to be mycoplasma-negative each month. Stassi Laboratory sphere cell lines were authenticated via short tandem repeat (STR) DNA profiling (GlobalFiler™ STR kit, Applied Biosystems) using an ABI PRISM 3130 genetic analyzer (Applied Biosystems) according to the manufacturer’s instructions. The STR profiles of CRC sphere cells were matched with those of their corresponding patient-derived tumors [48]. For CSphC differentiation, spheroids were cultured for 24 h in DMEM/F12 supplemented with 10% FBS, as described previously [40].

### Antibodies

The following primary antibodies were used for western blotting according to the manufacturer’s protocol: poly-(ADP-ribose)-polymerase (PARP) (Cell Signaling Technology Cat# 9542, RRID:AB_2160739), Bcl-2 (Cell Signaling Technology Cat# 15071, RRID:AB_2744528), β-Catenin (Santa Cruz Biotechnology Cat# sc-7963, RRID:AB_626807), β-actin (Santa Cruz Biotechnology Cat# sc-47778, RRID:AB_626632), GSK3β (Santa Cruz Biotechnology Cat# sc-81462, RRID:AB_1123754), GAPDH (Santa Cruz Biotechnology Cat# sc-47724, RRID:AB_627678), phospho-β-Catenin (Thr41-Ser45) (Thermo Fisher Scientific Cat# 702969, RRID:AB_2734823) and Lgr5 (Sigma-Aldrich Cat# HPA012530, RRID:AB_1849329), VDAC-1 (Santa Cruz Biotechnology Cat# sc-8829), p53-Ab (Santa Cruz Biotechnology cat#. sc-6243), phospo-p53-Ab (Cat#9289), BAX-Ab (Cat#2774) and acetyl-H3-Ab (Cat#9649) (Cell Signalling Technology); ATM (Cat# PC116) (Calbiochem); phospho-ATM-Ab (Abcam, Cat # Ab81292). The secondary antibodies used were as follows: polyclonal goat anti-rabbit IgG (H + L)-HRP conjugate (Bio-Rad Cat# 170-6515, RRID:AB_11125142) and polyclonal goat anti-mouse IgG (H + L)-HRP conjugate (Bio-Rad Cat# 1706516, RRID:AB_2921252), polyclonal rabbit anti-goat IgG-HRP conjugate (Santa Cruz Biotechnology Cat# sc-2768, RRID:AB_656964) and goat polyclonal secondary antibody against mouse IgG (H&L)-Alexa Fluor® 594 (Abcam Cat# ab150120, RRID:AB_2631447). Stem cell viability was evaluated using a 3D Cell Viability Assay (Promega) according to the manufacturer’s protocol.

### Drugs

VPA was purchased from Enzo Life Sciences and dissolved in water. A library of 17 epigenetic compounds for high-throughput screening was derived from the L1300-Selleck-FDA-Approved-Drug-Library from Selleck Chemicals. All the compounds, except VPA, were dissolved in dimethyl sulfoxide (DMSO) and provided at a concentration of 10 mM. Oxaliplatin (OXA; Accord), 5′-deoxy-5-fluorouridine, (5′-DFUR; Sigma‒Aldrich), capecitabine (CHT; Xeloda) and B20-4.1 (a monoclonal antibody against murine VEGF) were obtained from Roche. CHT is a prodrug that is activated by carboxyl esterase in the first metabolic step. However, owing to the low expression of carboxyl esterase in most cancer cell lines, all in vitro studies in cancer cells were performed with the CHT metabolite 5′-DFUR, which requires thymidine phosphorylase (TP) for conversion into the active drug 5-fluorouracil (5-FU), as previously noted [33]. BEV, a monoclonal antibody against human VEGF, was purchased from Aybintio. Stock solutions were diluted to the appropriate concentrations in the culture medium before they were added to the cells.

### Automated combinatorial drug screen and cell proliferation assay

Two thousand cells were seeded in low-attachment 384-well plates using a Microlab STAR dispenser (Hamilton, RRID:SCR_019993) (~ 30% confluence). After 24 h, the cells were treated with OXA plus 5′-DFUR (seven concentrations of each compound at threefold dilutions; the highest concentrations of OXA and 5′-DFUR were 400 nM and 16 µM, respectively) or medium as a control. Microlab STAR (Hamilton, RRID:SCR_019993) was used to assay the cells, 72 h later. Each cell type was treated with 17 epigenetic compounds either alone or in combination with the 7-point dilution series of OXA plus 5′-DFUR (the final concentrations are given in the Results section). A fixed dosage of the epigenetic drug was selected based on preliminary results. Cell viability was measured using the CellTiter-Glo 3D® Luminescent Assay kit (Promega). CellTiter-Glo reagent was added using a Microlab STAR (Hamilton, RRID:SCR_019993), and the cells were shaken and incubated for 10 min in the dark. The luminescence was measured using a Cytation 5 multimode Reader (Agilent-BioTek, RRID:SCR_99732). The luminescence was recorded by Gene5 Software (Agilent-Biotek, RRID: SCR_017317). Dose‒response curves were generated for both the monotherapy and combination therapy groups using Graph Pad Prism 7 to determine both growth rate-adjusted and traditional measures of drug sensitivity (areas under the curve (AUCs). We used the change in the AUC (ΔAUC) between the monotherapy and combination treatment curves as a proxy for synergy. For the cell proliferation assay with VPA plus OXA and/or 5′-DFUR, 100,000 cells/ml were plated in low-attachment multiwell plates and treated with the indicated drugs. The treatment doses and durations are described in the results section. Spheroids were scored using CellTiter-Glo® 3D Cell Viability Assay (Promega). Representative images were acquired at 40× magnification using the Opera Phenix High Content Screening System (HCS) (Revvity).

### Quantitative real-time PCR (qRT–PCR)

Total RNA was isolated from cells using TRIzol® total RNA isolation reagent (Gibco) according to the manufacturer’s recommendations. cDNA for qRT‒PCR analyses was synthesized using the QuantiTect Reverse Transcription Kit (Qiagen). The mRNA expression levels were quantified via the fluorescent SYBR Green method (Qiagen). Gene expression was measured using the 2^−ΔΔCT^ method and normalized to the level of the endogenous control B2M. The primers used in this study were as follows: B2M: forward, 5′-CTTCAGTCGTCAGCATGG-3′, reverse, 5′-GTTCTTCAGCATTTGGATTTC-3′; LGR5: forward, 5′- AATCCCCTGCCCAGTCTC-3′, reverse, 5′- CCCTTGGGAATGTATGTCAGA-3′; CK20: forward, 5′-TGTCCTGCAAATTGATAATGCT-3′, reverse, 5′-AGACGTATTCCTCTCTCACTCTCATA-3′; SURVIVIN: forward, 5′- GCCCAGTGTTTCTTCTGCTT-3′, reverse, 5′-GGACGAATGCTTTTTATG-3′; AXIN2: forward, 5′-CTCCTTATCGTGTGGGCAGT-3′, reverse, 5′-CTTCATCCTCTCGGATCTGC-3′; and *Ascl2:* forward, 5′-GGAAGCACACCTTGACTGGT-3′; reverse, 5′-GAAGTGGACGTTTGCACCTT-3′.

### Matrigel differentiation assay and immunohistochemistry

Two thousand cells were mixed with 70% Matrigel (Corning) and 30% medium, and 50 μl of the mixture was seeded as a droplet in a 12-well plate. After matrigel hardened (10 min at 37 °C), 1 ml of medium was added to the wells to cover the matrigel. Three days later, the medium was replaced with one supplemented with VPA or phosphate-buffered saline (PBS). After incubation as reported in the Results section, the CSphCs were embedded in paraffin as described previously [50]. Briefly, the CSphCs were fixed with 4% paraformaldehyde overnight at 4 °C. After dehydration, Matrigel samples were incubated in paraffin at 60 °C for 30 min and finally embedded in paraffin. Sections (5 µm thick) were cut from the paraffin-embedded blocks using a microtome, and the slides were stained for Lgr5 (dilution 1:300), CK20 (dilution 1:500), and β-catenin (dilution 1:200) via standard procedures according to the manufacturer’s protocols.

### Evaluation and quantification of the full-cell proteome

CSphCs were treated as previously reported or untreated and then lysed with 0.2% RapiGest SF (186002122, Waters) in 50 mM ammonium bicarbonate (AmB). Cells were lysed on ice for 2 h, denatured at 95 °C for 5 min in a Thermo mixer R (Eppendorf) at 300 rpm and subjected to three sonication cycles at a frequency of 20 kHz for 3 cycles until the viscosity of the sample was reduced. After centrifugation for 30 min at 14,000 rpm and 4 °C, the protein content was measured using the Bradford assay. Total proteins (20 µg) was reduced with 10 mM dithiothreitol (DTT) and then alkylated with 24 mM iodoacetamide (IAA). Afterward, protein digestion was performed by diluting the samples at a 1:1 v/v ratio with 0.1% RapiGest SF and 1:50 ratio w/w sequencing grade trypsin (V5111, Promega). The next day, the RapiGest SF was removed according to the manufacturer’s instructions, and then the samples were desalted using a C18 stage tip (Millipore, Merck KGaA) and dried under vacuum. The lyophilized desalted fractions were resuspended in 20 µL of 0.1% trifluoroacetic acid (TFA) and were processed using liquid chromatography–tandem mass spectrometry (LC‒MS/MS) proteomic technology as described by Iannelli et al. [37]. In detail, about 2.5 μg of protein was injected into a Dionex UltiMate 3000 Rapid Separation LC nanosystem (Thermo Fischer Scientific) coupled with an AmaZon ETD mass spectrometer (Bruker Daltonics). Progenesis QI for proteomics v. 4.2 software (Nonlinear Dynamics) was used for label-free quantification.

### Immunoprecipitation and LC-MS/MS analysis

CSphCs #08 were plated and treated with or without VPA (0.5 mM). After 24 h, the cells were collected, and protein lysates were obtained by digestion with 0.1% NP-40 buffer. Then, the total cell lysates (about 500 µg) were immunoprecipitated with β-catenin antibody (Santa Cruz Biotechnology Cat# sc-7963, RRID:AB_626807) (10 µL/sample) and Protein A Mag Sepharose (GE28-9670-70) overnight at 4 °C according to the manufacturer’s instructions (GE Healthcare). The next day, the magnetic beads were washed three times with 50 mM AmB and 400 ng of sequencing grade trypsin (V5111, Promega) in 100 µL of 0.1% RapiGest SF (186002122, Waters) in 50 mM AmB was added, and the mixture was incubated for 30 min at 37 °C. Next, 10 mM DTT was added to the supernatant (100 µL) for 1 h of incubation at 37 °C for reduction, and then 24 mM IAA was added for 1 h of incubation at 37 °C for alkylation. Finally, 200 ng of sequencing grade trypsin was added to the mixture, which was subsequently incubated overnight at 37 °C. The next day, the RapiGest SF was removed, and the sample was desalted using a C18 stage tip (ZTC18S960, Millipore) and dried under vacuum. The lyophilized desalted fractions were resuspended in 20 µL of 0.1% TFA before injection onto a Dionex UltiMate 3000 Rapid Separation LC nanosystem (Thermo Fischer Scientific) coupled with an AmaZon ETD mass spectrometer (Bruker Daltonics). Protein identification was performed using the Mascot search engine software (Matrix Science) ver. 2.8.

### Protein network analyses

The differentially expressed proteins were identified by MS and analyzed using Ingenuity Pathway Analysis (IPA) software (GeneGo Inc., RRID:SCR_008653). The IPA software includes a manually annotated database of protein interactions and metabolic reactions obtained from the scientific literature. The networks were graphically illustrated as hubs (proteins) and edges (relationships with proteins).

### Fold change analysis

The fold change was calculated based on the protein expression levels in the treated samples and the control. The heatmap was generated using the TMEV software [51].

### TCF/LEF reporter assay

CSphCs #147 were transiently cotransfected with a TCF/LEF luciferase reporter vector and a construct that constitutively expressed Renilla luciferase using Lipofectamine 2000 (Thermo Fisher Scientific) according to the manufacturer’s instructions. After 48 h of transfection, the CSphCs were treated as previously reported, and the luciferase activity was measured 24 h later using the Dual Luciferase® Reporter Assay System (Promega).

### Immunofluorescence

Single cells (6000/well) were plated in 96-well plates and treated as indicated in the figure legends. Then, the cells were fixed in 4% paraformaldehyde (20 min at room temperature (RT)), blocked with 0.2% PBS/bovine serum albumin solution (5 min at RT) and incubated with the primary β-catenin antibody (Santa Cruz Biotechnology Cat# sc-7963, RRID:AB_626807) (1:600) or γH2A× antibody (Millipore Cat# 05-636, RRID:AB_309864) (1:1000) for 1 h at 37 °C. After washing, the cells were incubated with anti-rabbit Alexa Fluor 595 (Thermo Fisher Scientific) for 1 h at RT. The cells were subsequently washed and incubated for 5 min with 4′,6-diamidino-2-phenylindole (DAPI; Thermo Fisher Scientific). Representative images were acquired with an Opera Phenix High Content Screening system (Revvity) at 40× magnification, and image quantification was performed using Harmony software (Revvity). For F-actin fiber costaining, after β-catenin staining, the cells were stained with rhodamine phalloidin (Promega) according to the manufacturer’s recommendations.

### Western blotting

Western blotting was performed according to standard procedures as described previously [52]. Images were acquired with an Image Quant LAS 500, and the intensity was measured using Image Quant TL image software (GE Healthcare).

### Limiting dilution assay

CSphCs were cultured as single cells with or without VPA. After 24 h, the cells were collected and plated in serial dilutions (1, 2, 4, 8, 16, 32, 64, and 128 cells/well) in low-attachment flat-bottom 96-well microplates using a fluorescence-activated cell sorting (FACS) Melody sorter (BD Biosciences). Stem cell frequency was evaluated after three weeks via extreme limiting dilution analysis as previously described [36].

### Apoptosis analysis

CSphCs were treated with VPA or chemotherapy alone or in combination, as reported in the figure legends. CSphCs were dissociated and stained with an anti-CD133-APC antibody (1:100, Miltenyi Biotec, # 170-076-513, RRID:AB_2802082) in PBS for 20 min at 4 °C, washed with PBS and resuspended in PBS-annexin V-FITC (BD Biosciences) for 15 min at 4 °C to identify apoptotic cells. Flow cytometry analysis was performed using the FACSCanto system (BD Biosciences).

### Healthy mouse organoid culture

Organoid cultures were generated from the first 5 cm of the duodenum. The intestine was first flushed with cold PBS and then cut longitudinally with the villi facing upward. After careful removal of the villi using a glass coverslip, the intestine was sliced into small pieces, washed 20 times with cold PBS and dissociated with 2 mM EDTA at 4 °C for 30 min. Then, ~100–200 crypts were resuspended in 25 μl of Matrigel (BD Bioscience) per well in a 48-well plate and maintained in Advanced DMEM/F12 (Life Technologies) supplemented with 100 ng/ml murine Noggin (Prospec), 50 ng/ml murine endothelial growth factor (PeproTech), and 500 ng/ml R-Spondin (R&D Systems). Three days after plating, the organoid cultures were treated with 0.5 mM VPA in media for 24 h.

### In vivo xenograft studies

All animal procedures were performed in accordance with ARRIVE guidelines [53] (European directive 2010/63/UE and Italian Law—Directive 2010/63/EU and DL 26/2014) and were approved by the appropriate institutional review board (no. 377/2018-PR). Five-week-old female nude athymic mice (Envigo) were used for the CSphC #08, CSphC #147, and CT26 xenograft models. The mice were acclimatized to the Animal Care Facility of the Laboratory of Mercogliano (AV) INT-IRCCS “Fondazione G. Pascale”. After one week, single CSphCs (2 × 10^5^) suspended in 200 μl of PBS were injected subcutaneously (s.c.) into the flank regions of the mice. The mice were randomized based on the random number generator function in Microsoft Excel (RRID:SCR_016137) in different experimental groups (9 mice/group) to receive intraperitoneally (i.p.) with VPA (dissolved in water and diluted in physiological saline), OXA (dissolved in water and diluted in physiological saline), CHT (dissolved in 40 mM citrate buffer, pH 6, containing 5% arabic gum solution) or an anti-VEGF moAb (B20-4; dissolved in water and diluted in physiological saline), as noted in Fig. 5. Mice in the control groups were treated with physiological saline or DMSO plus physiological saline at a ratio of 1:1. Sample size was based on estimations by power analysis with a level of significance of 0.05 and a power of 0.8 (G*Power version 3.1.9.7) (RRID:SCR_013726). The tumor volume and percentage change in tumor volume in the experimental groups were compared with those in the vehicle control groups. Overall survival was considered a positive event in the Kaplan–Meier curves analysis. Humane endpoint cutoffs were defined as a tumor volume of 1500 cm^3^ and a weight reduction of 20% reduction.

### Statistical analysis and cartoon generation

All the experiments were performed at least three times. Statistical significance was determined by one-way ANOVA, Tukey’s multiple comparison test, Dunn’s multiple comparisons test and the log rank test; *p* < 0.05 was considered to indicate statistical significance. The assumption of equal variances was tested and met for all statistical comparisons. All the statistical evaluations were performed using GraphPad Prism 7 (RRID:SCR_002798). All the cartoons were created using BioRender.com (RRID:SCR_018361).

## Supplementary information

Supplementary figures 1-11

Uncropped western blots

## Data Availability

The raw data generated in this study are publicly available in Zenodo (RRID:SCR_004129) (10.5281/zenodo.14380040). All datasets are available from the corresponding author on reasonable request.
