# Supplementary material for: Valproic acid improves the efficacy of oxaliplatin/fluoropyrimidine-based chemotherapy by targeting cancer stem cell via β-Catenin modulation in colorectal cancer
Source: Cell Death Dis. 2025 Aug 1;16(1):583. doi: 10.1038/s41419-025-07902-8 (PMC12316941; doi:10.1038/s41419-025-07902-8)
Supplement: Supplementary file 1 — Supplementary figures 1-11 [file 41419_2025_7902_MOESM1_ESM.pdf]

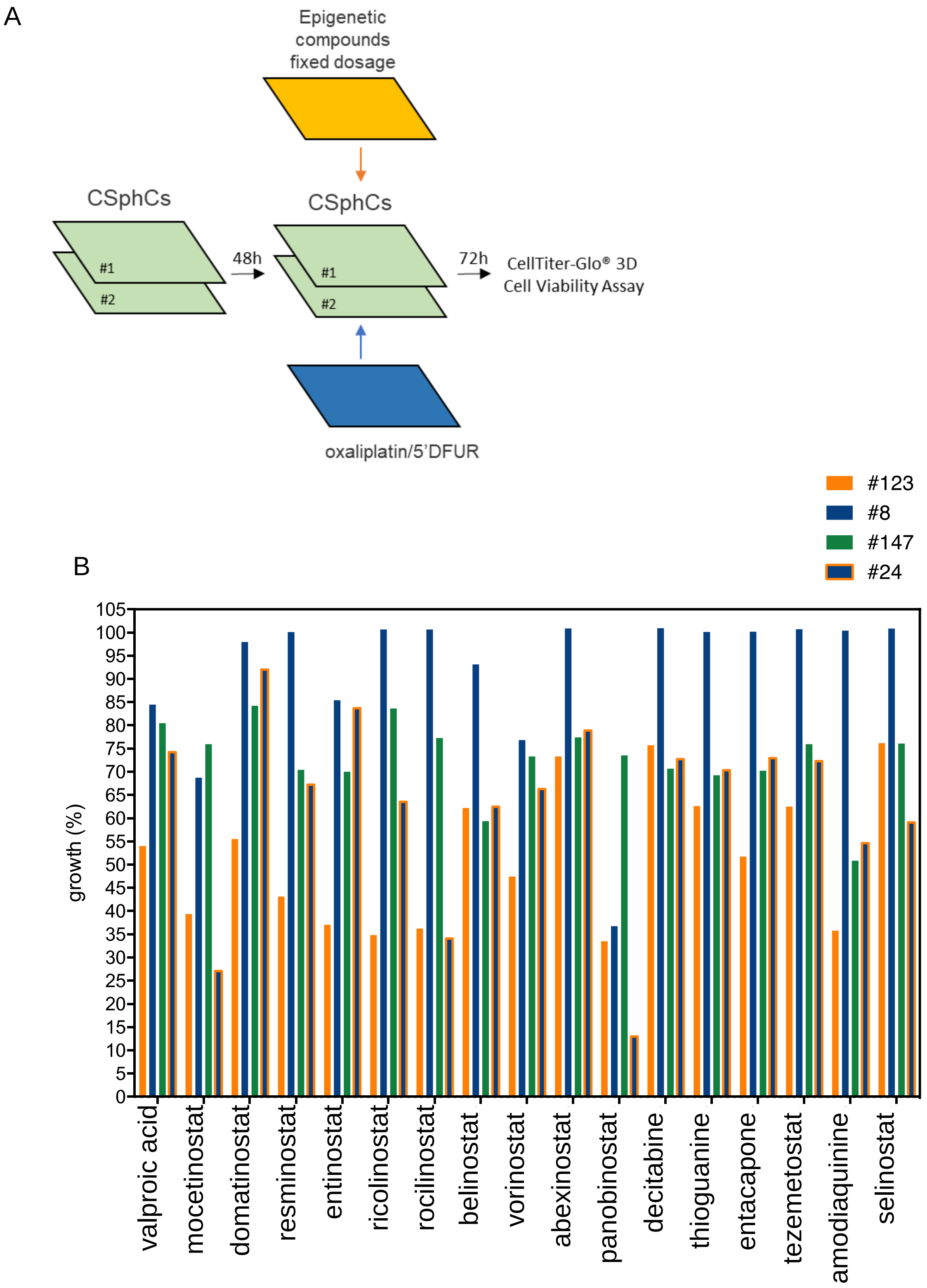

Supplementary Figure 1. Anti-proliferative effects of epigenetic compounds in combination with OXA/5'-DFUR in CR-CSphC. (A) Schematic representation of drug screening performed based on combination of OXA/5′-DFUR chemotherapy in escalation doses plus the epigenetic coumpond administred at a fixed dose. The cells were plated (600cells/well) in 384-well and after 48 hours were treated with epigentic coumpond and after 72 hours the cell growth was measured by CellTiter-Glo 3D® Luminescent Assay (see Materials and Methods). (B) The cell growth evaluated in the previous described screening for all 17 epigenetic coumpons administred as single drug (valproic acid 0.5mM, mocetinostat 1 µM, domatinostat 1 µM, resminostat 1 µM, entinostat 1 µM, ricolinostat 1 µM, rocilinostat 1 µM, belinostat 1 µM, vorinostat 1 µM, abexinostat 1 µM, panobinostat 0.1µM, decitabine 1 µM, thioguanine 1 µM, entacapone 1 µM, tezemetostat 1 µM, amodiaquinine 1 µM, selinostat 1 µM) , as reported in graph.

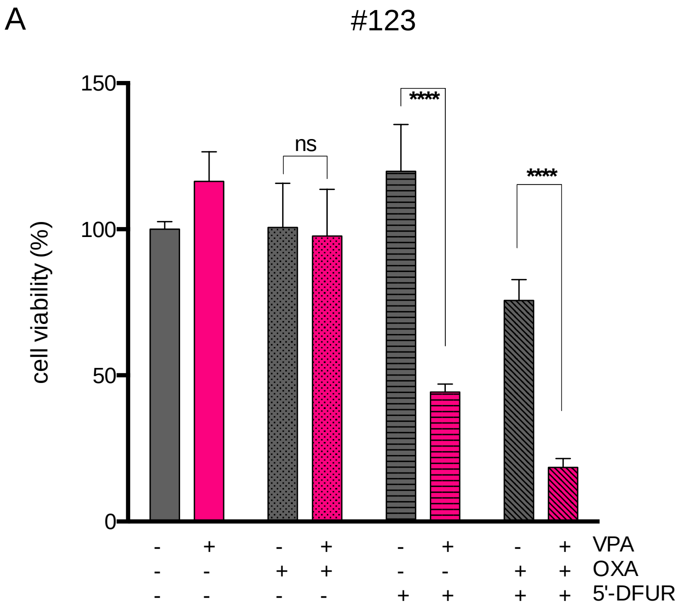

Supplementary Figure 2. Anti-proliferative effects of VPA in combination with OXA/5’-DFUR in CR-CSphC. (A) CR-CSphC #123 were plated (4000 cells/well) in 96-well and after 48 hours were treated with VPA (0.5mM) and/or with oxaliplatin (200 nM) and/or 5’-DFUR (4µM) and after 72 hours the cell growth was measured by CellTiter-Glo 3D® Luminescent Assay (see Materials and Methods).

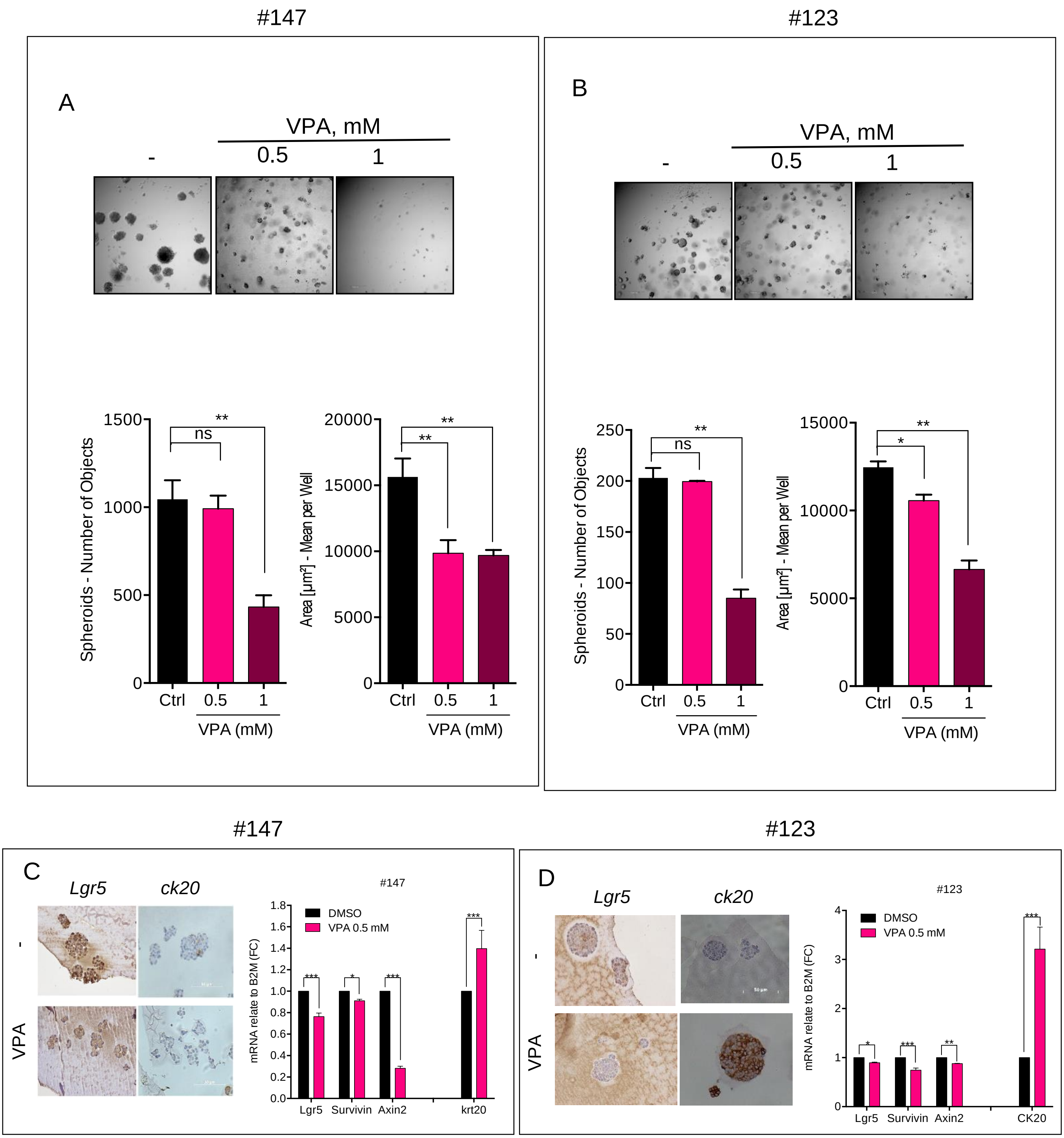

Supplementary Figure 3. VPA treatment of 0.5mM for 24h induced a reduction of number and size of in CR-CSphCs #147 **(A)** and #123 **(B)**, growing in 3D matrigel matrix. The images and quantification analysis were by Opera Phoenix microscope and Harmony software. A short-term VPA treatment revealed marked morphological changes, the CR-CSphCs treated with VPA (0,5mM) formed distinct polarized structures. The change in morphology of spheroids upon VPA treatment (16h- 0,5 mM) were associated with increased expression of differentiation marker cytokeratin 20 (CK20) and a significant drop down of stem cell markers (Lgr5, Survivin and Axin2) at mRNA levels **(C)** and at protein levels as showed by IHC staining **(D).**

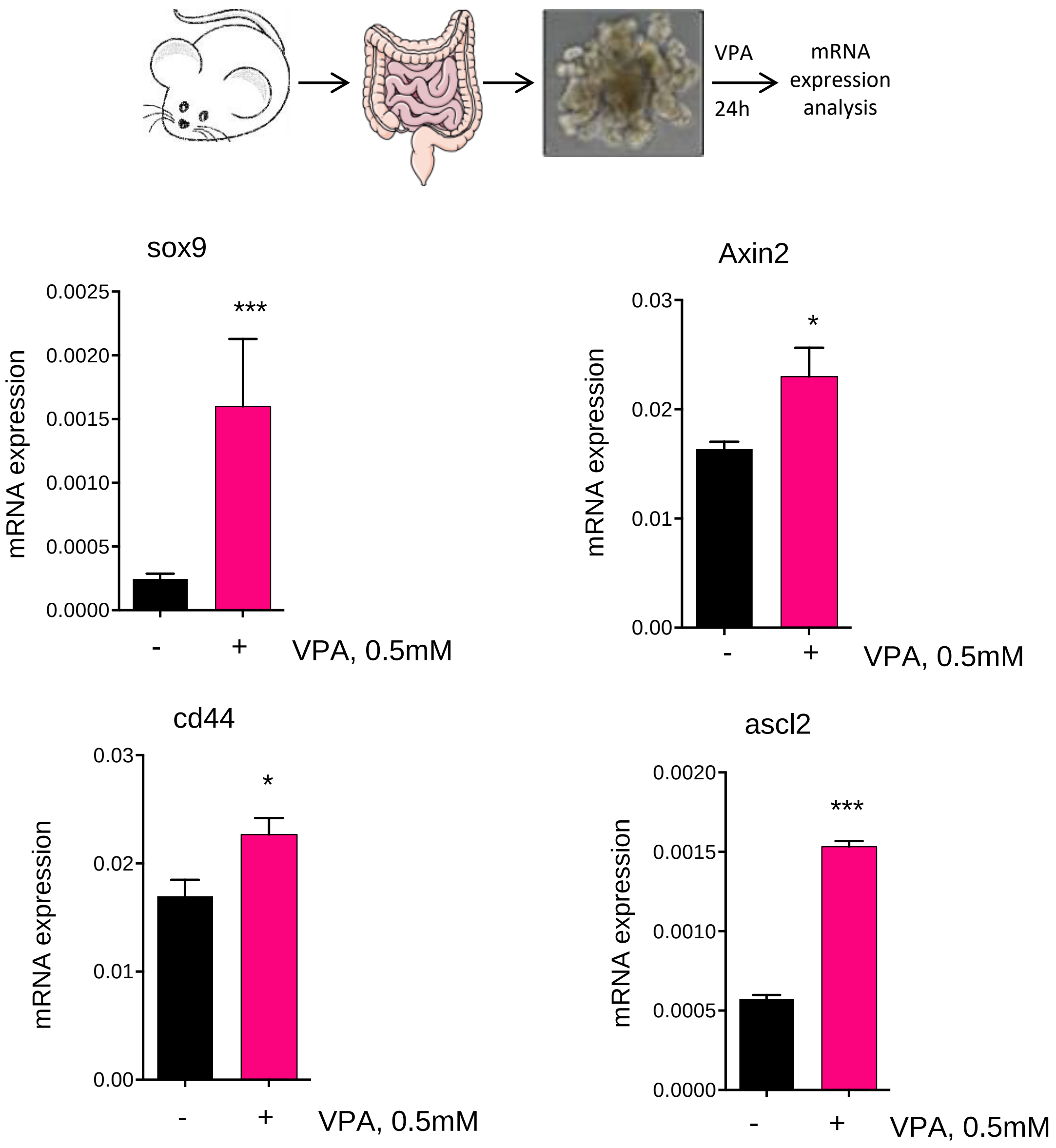

Supplementary Figure 4. VPA treatment results in an opposite effect in healthy organoids. Organoids are isolated from small intestine of healthy mice and cultured in vitro in matrigel. After 24h of VPA treatment 0.5mM, the organoids are isolated and mRNA expression analysis of Sox9, CD24, Axin2, Ascl2 stem markers is performed. B2M gene expression is used to normalize. (Statistically significant results by t-test are reported (*** indicates P<0.0001, ** indicates P<0.001, * indicates P<0.05).

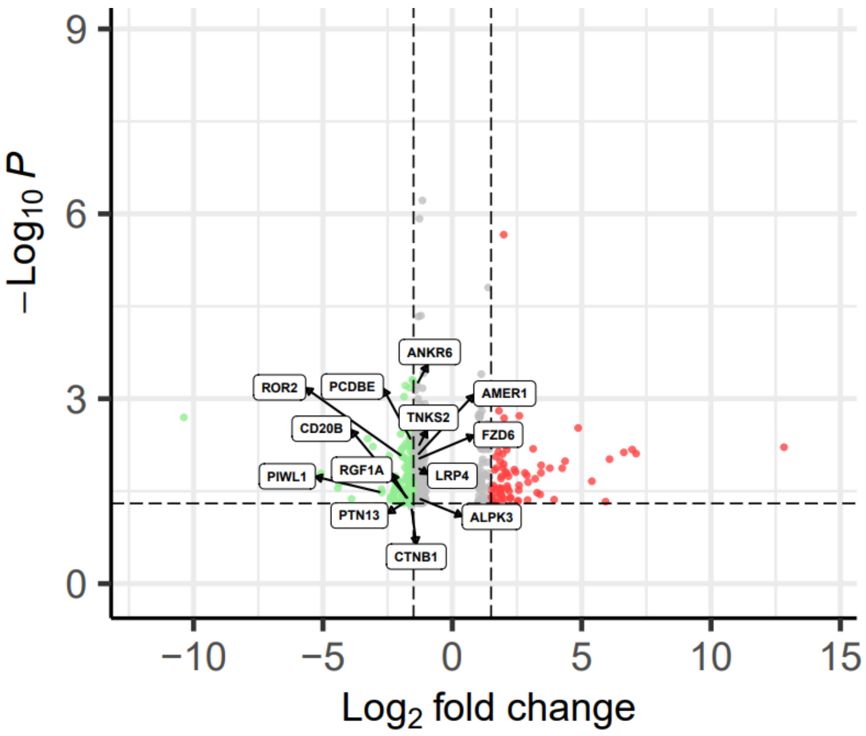

Supplementary Figure 5. VPA modulates established stem cell-associated genes in Mass Spectrometry data. Volcano plot shows the up-regulated (red) and down-regulated (green) proteins in CSphC#08 upon VPA treatment. The criteria were a P value less than 0.05 and a fold change of less than +1 or -1 in one experimental group.

A

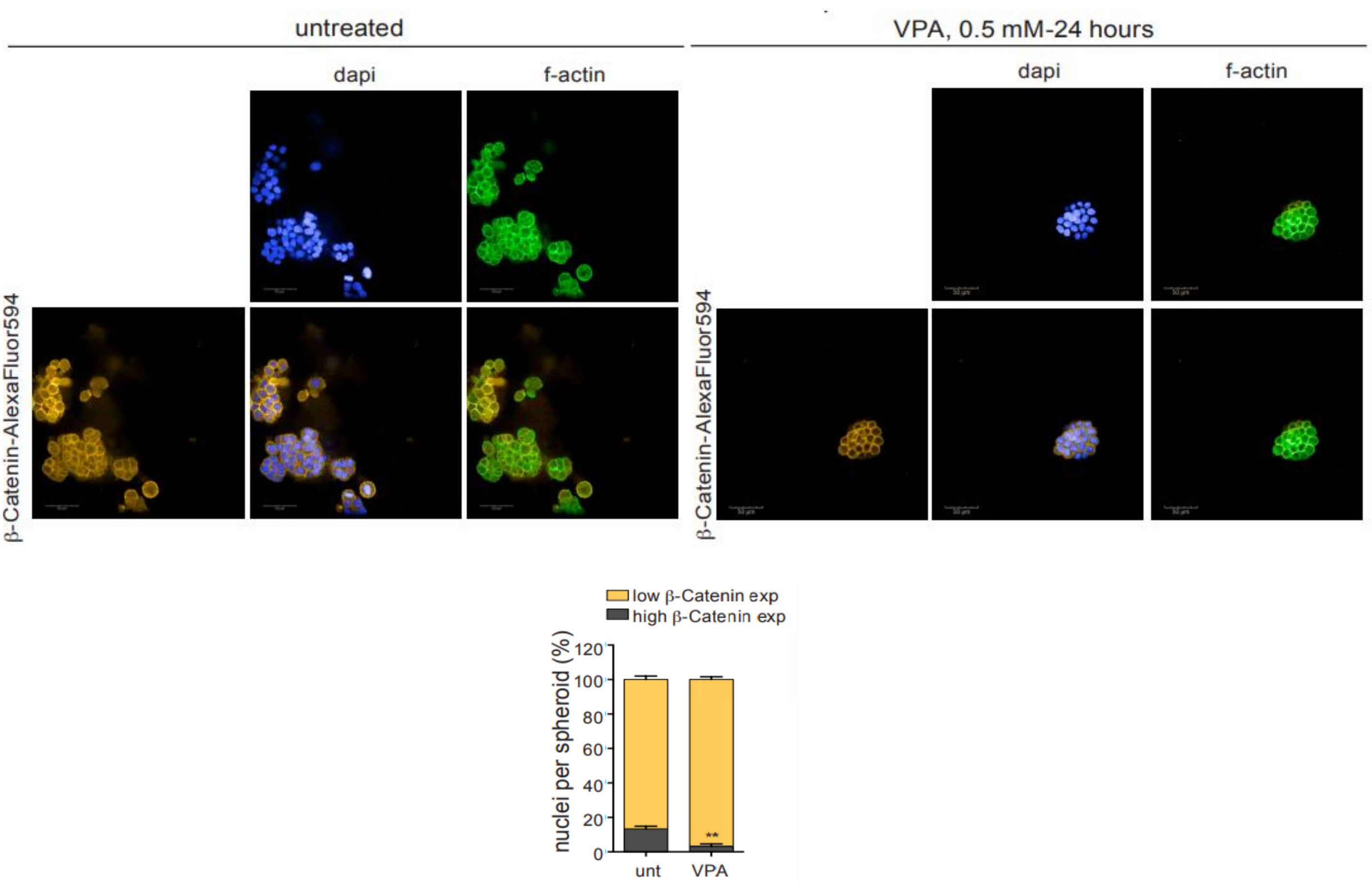

B

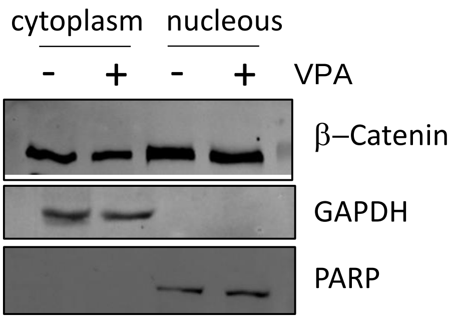

Supplementary Figure 6. VPA modulates Wnt signalling pathway and β-catenin expression.
(A) A representative overview of confocal images displaying b-Catenin expression, f-actin fibers (stained with falloidine-FITC) and nucleus (stained by DAPI) in CSphCs #147 untreated and treated with VPA 0.5mM, magnification 40X. Scale bar: 50 μm. b-Catenin-nuclear intensity is quantified by Harmony software for each cell in each single spheroid and then clustered in b-Catenin low expression nucleus and in b-Catenin high expression nucleus, considering the average of b-Catenin intensity in each spheroid. Error bars represent s.e.m. (spheroids n= 30); the reported experiment is representative of three independent experiments. (B) Protein expression in cytoplasm and nucleous of b-Catenin in CSph#147 cells treated with VPA 0.5mM, collected at 6 hours and evaluated by western blot. GAPDH and PARP serves as loading control Statistically significant results by t-test are reported (*** indicates P<0.0001, ** indicates P<0.001, * indicates P<0.05).

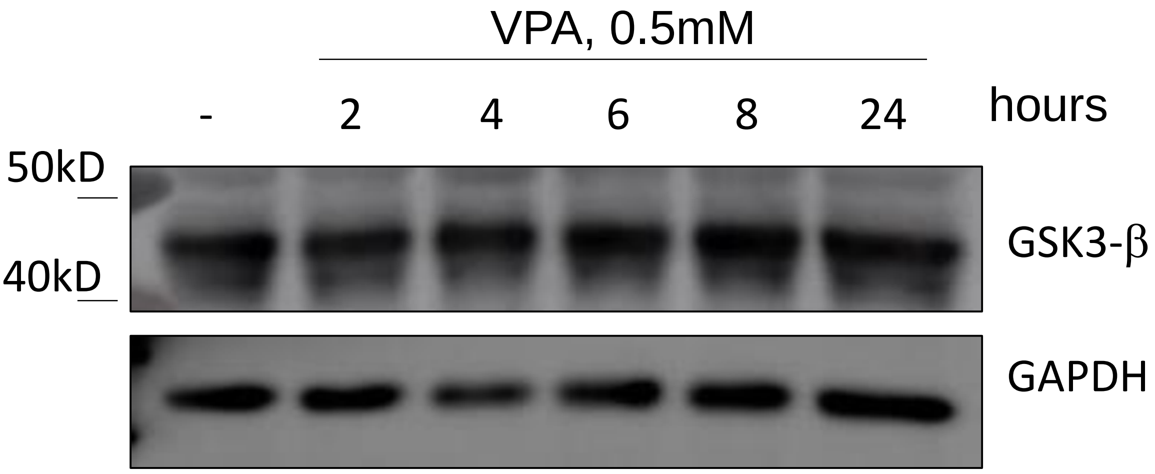

Supplementary Figure7. VPA effects on GSK3β.
Protein expression of GSK3-β 2AX in CSph#08 cells treated with VPA 0.5mM, collected at 2, 4, 6 ,8 and 24 hours and evaluated by western blot. GAPDH serves as loading control.

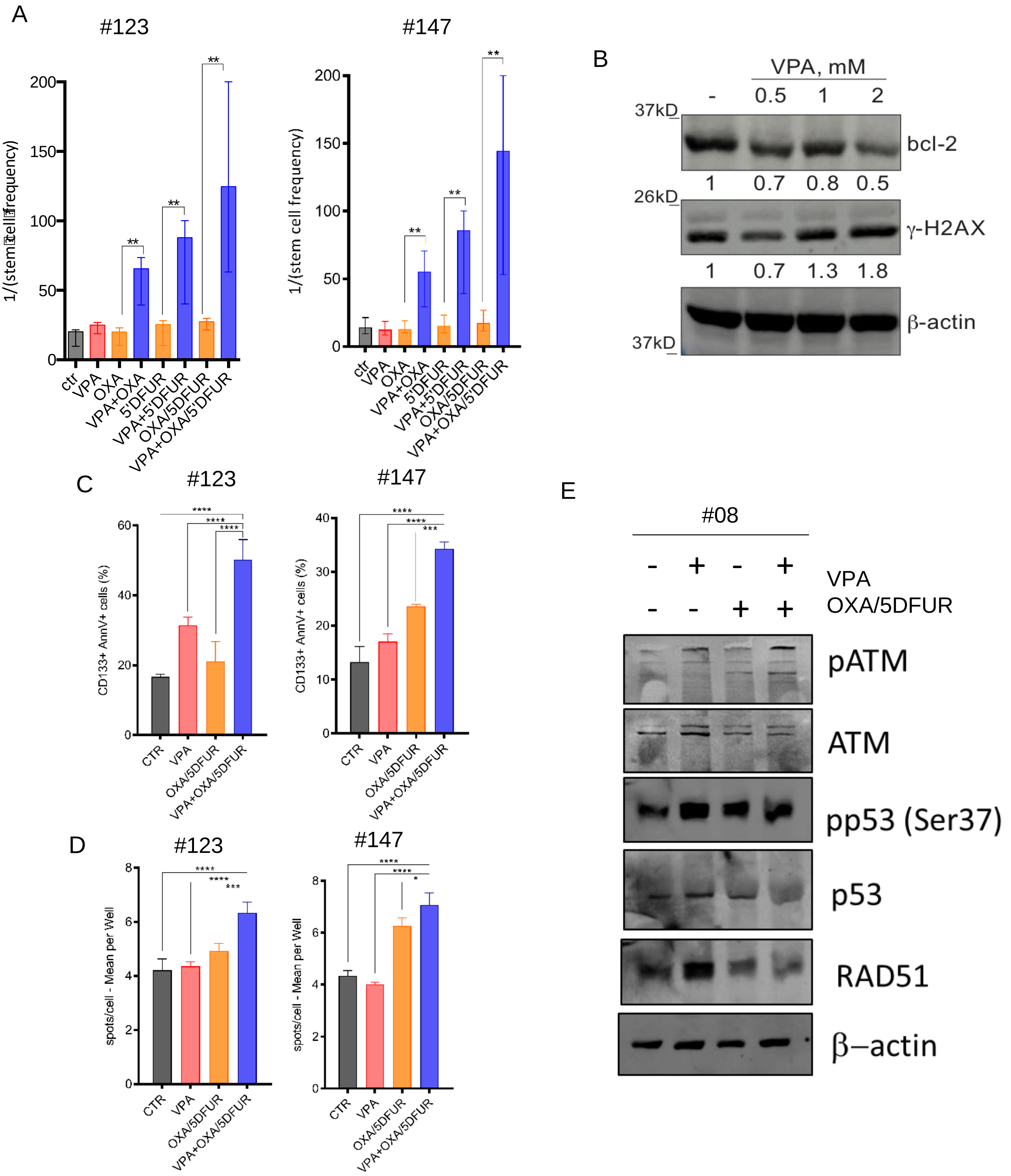

Supplementary Figure 8. In all experiments the used dosage are VPA, 0.5mM, oxaliplatin, 100 nM and 5'-DFUR, 4 mM. (A) Limiting dilution assay performed on CSphCs #123 and #147, untreated or treated for 24 h with VPA plus oxaliplatin and 5'-DFUR and plated in ultra-low 96-well without additional treatment for three weeks. Clonal frequency was evaluated with the Extreme Limiting Dilution Analysis 'limdil' function as described in Material and Methods section. (B) Western blot analysis of bcl-2 and γH2AX protein levels upon VPA 0.5, 1 and 2 mM, administered for 24h in CSphCs #147. (C) Apoptosis was evaluated by Caspase 3/7 activity assay in CSphCs #123 and #147 treated as previously. (D) DNA damage was analyzed in CSphCs #123 and #147 by visualizing double strand break marker γH2AX foci. CSphCs #123 and #147 treated for 24 h with or without VPA and oxaliplat/5'-DFUR. CSphCs are fixed, stained for γH2AX (orange) and DAPI for nuclei (blue) and measured by Opera Phenix confocal microscopy. The number of spot for each cell is analyzed by Harmony software. (E) Western blot analysis of pATM, ATM, pp53, p53 and RAD51 protein levels upon VPA 0.5 5DFUR 1uM and oxaliplatin 50uM, administered for 24h in CSphCs #08. b-actin is used as loading control. *** indicates P<0.0001, ** indicates P<0.001, * indicates P<0.05).

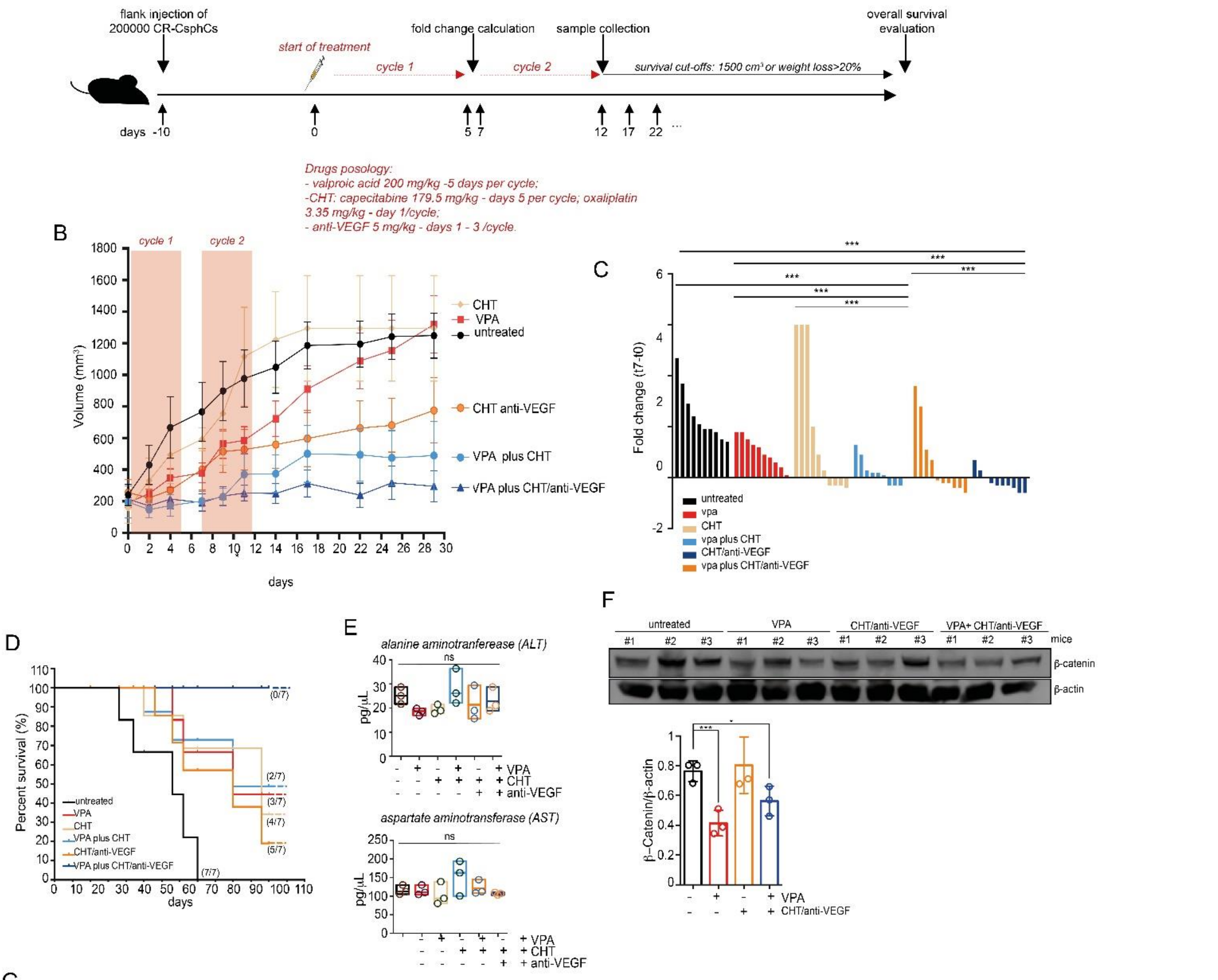

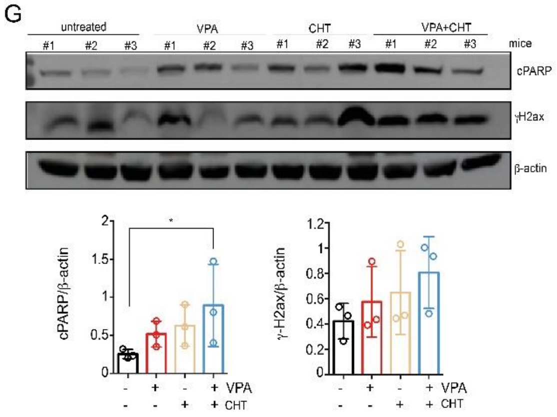

Supplementary Figure 9. A schematic view of in vivo xenograft experiment with timing and agents concentration. CSphC #147 are s.c. injected into athymic mice as described in the Materials and Methods. When established tumors were palpable, mice were treated as reported in figure. The mice are sacrificed when the tumor volume becomes higher then 1500 mm^3 and/or when the weight lost is higher then 20%. (B) Tumor volume curves for CSphC#08. For each single or combination of treatment mean ± SEM are reported at specified time points. Statistically significant results by Anova are reported in black for comparison of untreated, VPA, CHT and VPA+CHT groups and in red for comparison of untreated, VPA, CHT/anti-VEGF and VPA+ CHT/anti-VEGF groups. (C) The synergistic effect is already observed upon 1 cycle of treatment as showed by waterfall plot of the fold-change in tumor volume compared with baseline (day 0-start of treatment) of transplanted tumors. P values are calculated using a two-tailed, unpaired Student's t-test. (*** indicates P<0.0001, ** indicates P<0.001, * indicates P<0.05). (D) Kaplan–Meier curves comparing the survival of single and combination groups of treatment. The number of mice per treatment condition is indicated. (E) Serum alanine-aminotransferase (ALT) and aspartate aminotransferase (AST) amount is evaluated in 3 mice for each group at the end of two cycle of treatment. (F) Protein expression of β-Catenin in CSph#147 tumor samples collected at the end of treatment, evaluated by western blot. Quantification and fold change to untreated are reported in graph. (G) Protein expression of PARP cleaved and γH2AX in CSph#147 tumor samples collected at the end of treatment, evaluated by western blot. Quantification and fold change to untreated are reported in graph. β-actin serves as loading control. (*** indicates P<0.0001, ** indicates P<0.001, * indicates P<0.05).

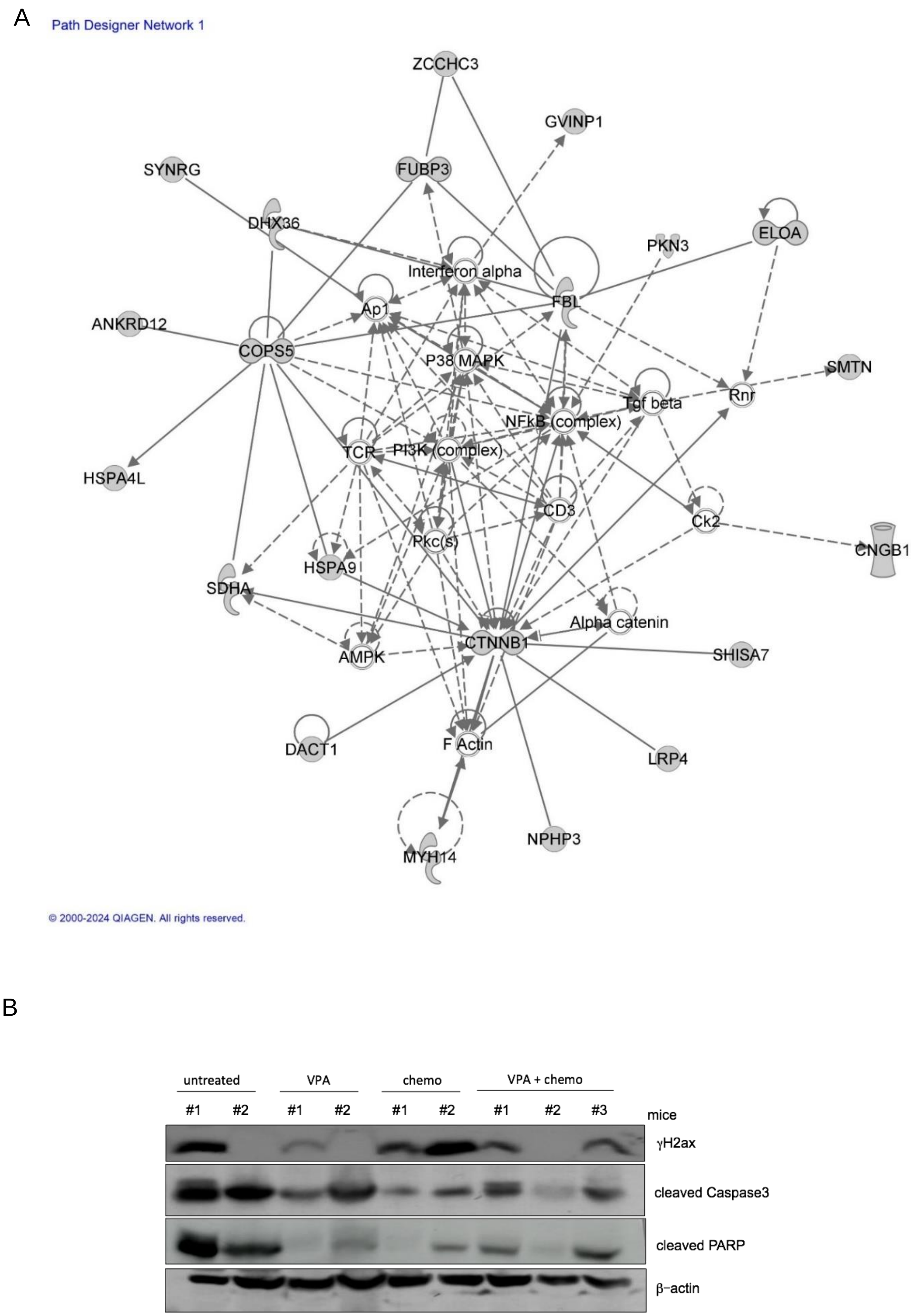

Supplementary Figure 10. (A) Visual representation of the network generated by IPA. The Ingenuity® Pathway Analysis networks legend is at http://qiagen.force.com/KnowledgeBase/articles/ Basic_Technical_Q_A/Legend. (B) Protein expression of PARP cleaved, Caspase 3 cleaved and γH2AX in CSph#08 tumor samples collected at the end of treatment, evaluated by western blot. Quantification and fold change to untreated are reported in graph. β-actin serves as loading control.

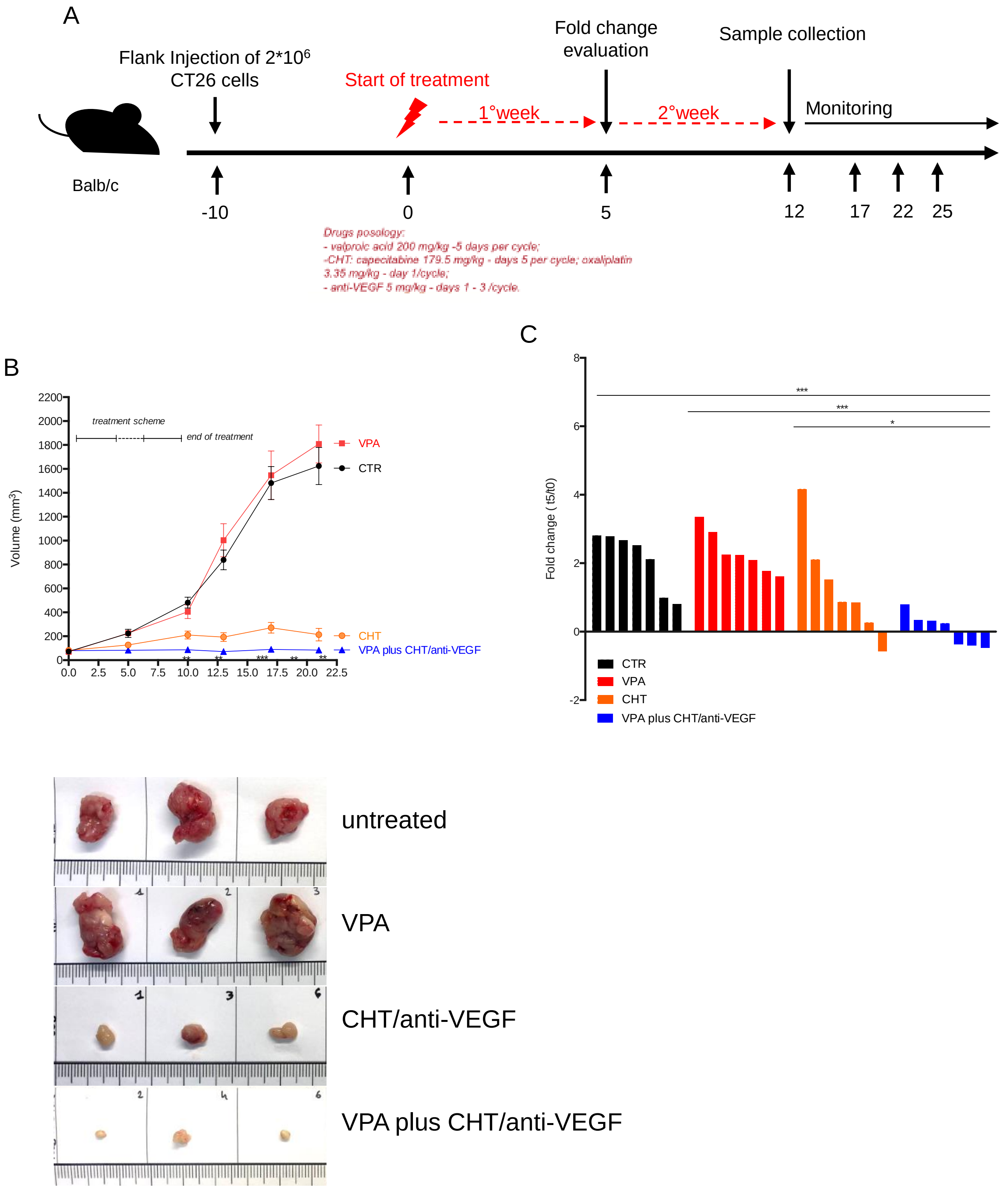

Supplementary Figure 11. A schematic view of in vivo xenograft experiment with timing and agents concentration. CT26 are s.c. injected into athymic mice as described in the Materials and Methods. When established tumors were palpable, mice were treated as reported in figure. (B) Tumor volume curves for CT26. For each single or combination of treatment mean ± SEM are reported at specified time points. Statistically significant results by Anova are reported. (C) The synergistic effect is already observed upon 1 cycle of treatment as showed by waterfall plot of the fold-change in tumor volume compared with baseline (day 0-start of treatment) of transplanted tumors. P values are calculated using a two-tailed, unpaired Student's t-test. (*** indicates P<0.0001, ** indicates P<0.001, * indicates P<0.05). (D) Representative images of collected tumor tissue collected at the end of treatment are showed.
