# Supplementary material for: Valproic acid improves the efficacy of oxaliplatin/fluoropyrimidine-based chemotherapy by targeting cancer stem cell via β-Catenin modulation in colorectal cancer
Source: Cell Death Dis. 2025 Aug 1;16(1):583. doi: 10.1038/s41419-025-07902-8 (PMC12316941; doi:10.1038/s41419-025-07902-8)
Supplement: Supplementary file 2 — Uncropped western blots [file 41419_2025_7902_MOESM2_ESM.pptx]

## Slide 1
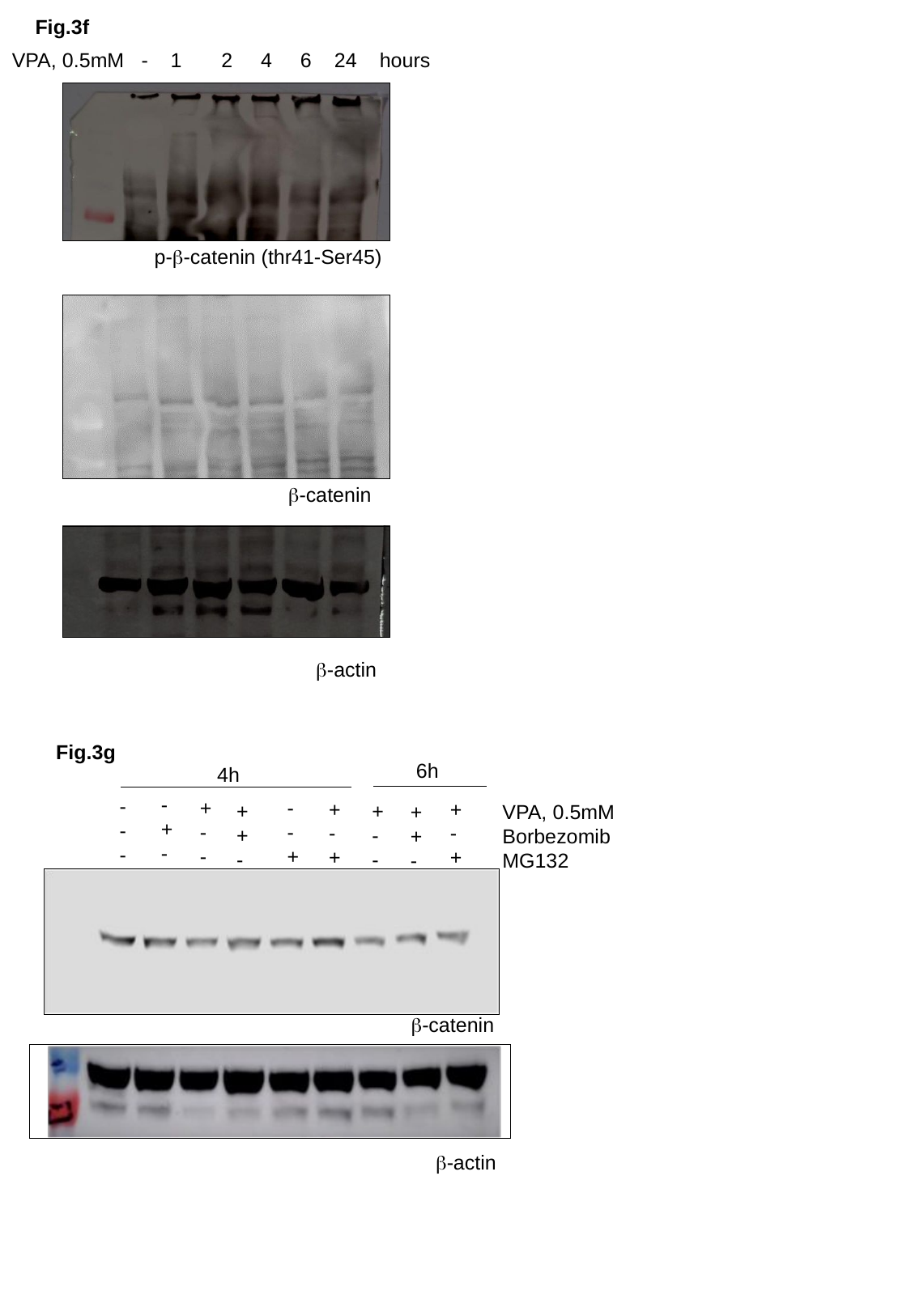

Fig.3f
VPA, 0.5mM - 1 2 4 6 24 hours
p-b-catenin (thr41-Ser45)
b-catenin
b-actin
Fig.3g
6h
4h
-
+
-
-
-
-
+
-
-
-
-
+
+
-
+
+
-
+
+
+
-
+
-
-
+
+
-
VPA, 0.5mM
Borbezomib
MG132
b-catenin
b-actin

## Slide 2
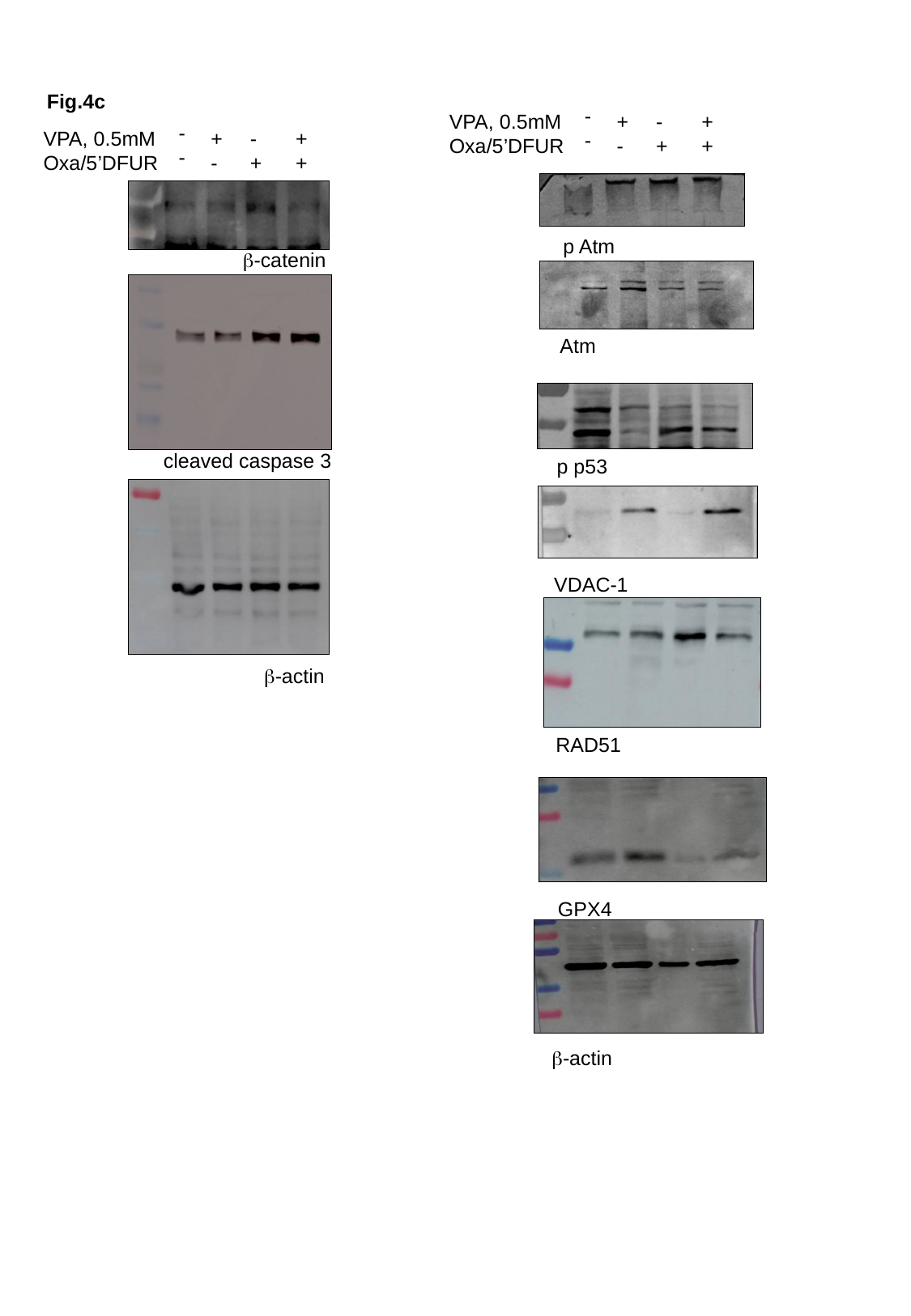

Fig.4c
-
-
VPA, 0.5mM
Oxa/5’DFUR
+
-
-
+
+
+
-
-
VPA, 0.5mM
Oxa/5’DFUR
+
-
-
+
+
+
p Atm
b-catenin
Atm
cleaved caspase 3
p p53
VDAC-1
b-actin
RAD51
GPX4
b-actin

## Slide 3
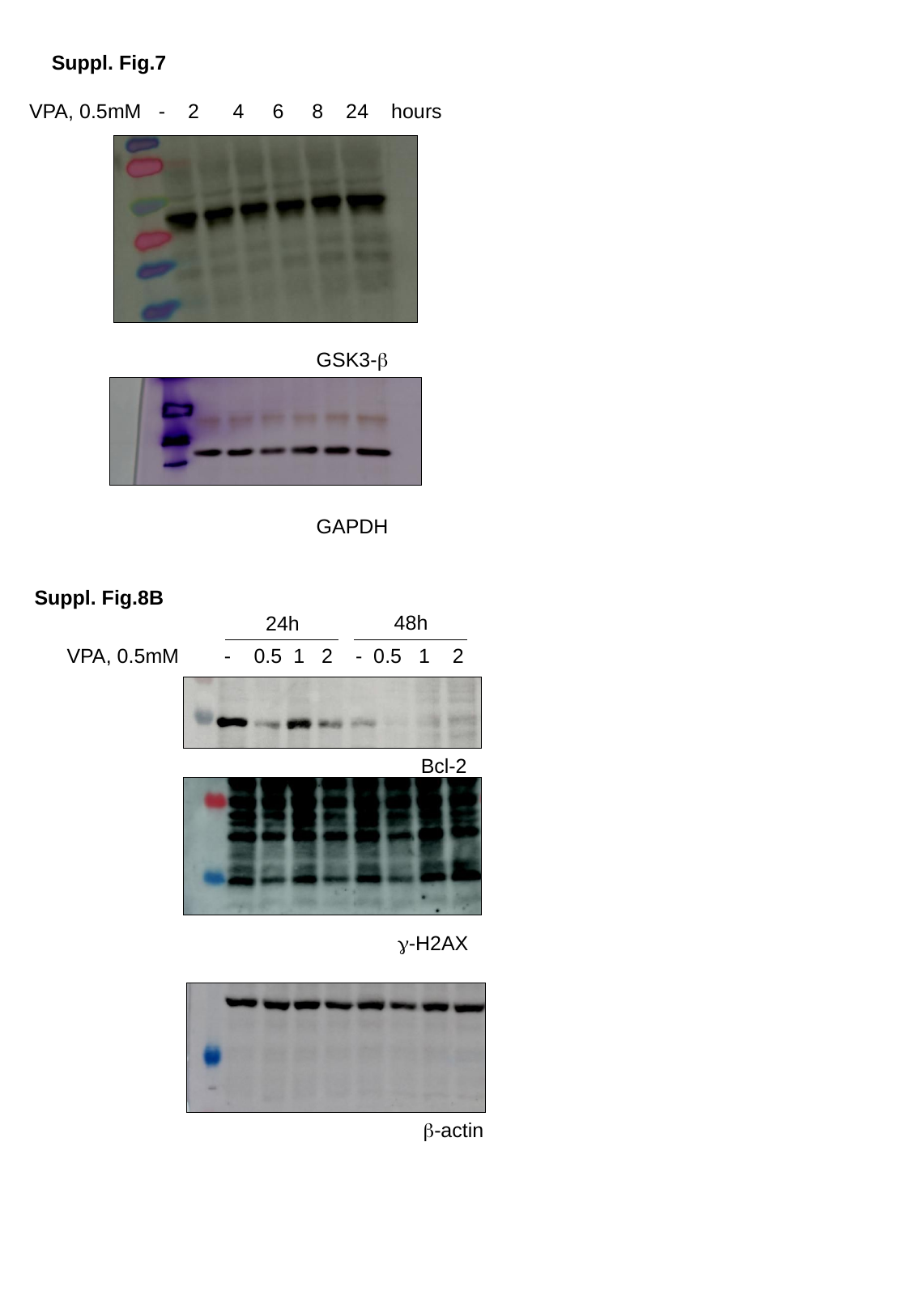

Suppl. Fig.7
VPA, 0.5mM - 2 4 6 8 24 hours
GSK3-b
GAPDH
Suppl. Fig.8B
48h
24h
VPA, 0.5mM - 0.5 1 2 - 0.5 1 2
Bcl-2
g-H2AX
b-actin

## Slide 4
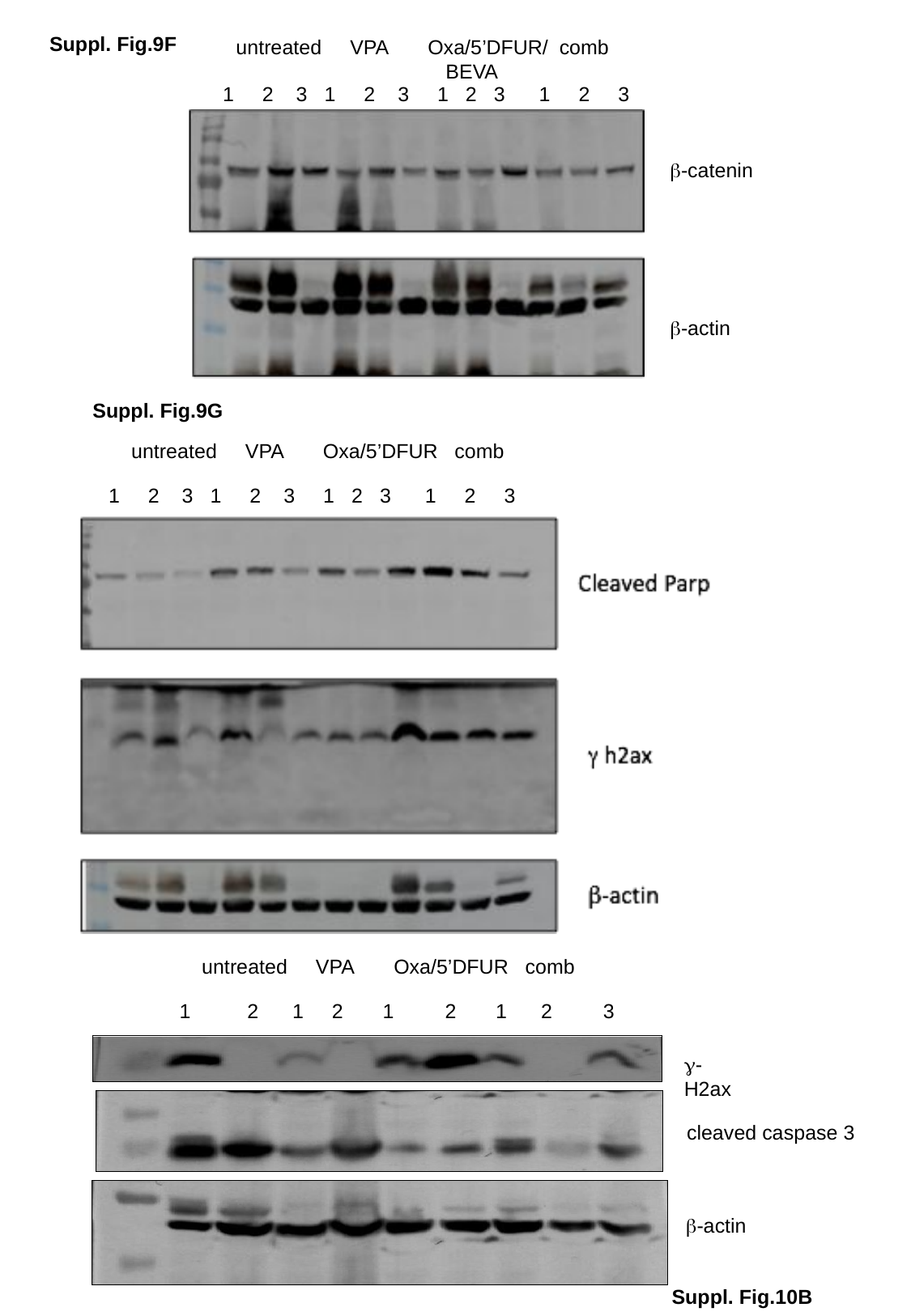

Suppl. Fig.9F
 untreated VPA Oxa/5’DFUR/ comb
 BEVA
1 2 3 1 2 3 1 2 3 1 2 3
b-catenin
b-actin
Suppl. Fig.9G
 untreated VPA Oxa/5’DFUR comb
1 2 3 1 2 3 1 2 3 1 2 3
 untreated VPA Oxa/5’DFUR comb
1 2 1 2 1 2 1 2 3
g-H2ax
cleaved caspase 3
b-actin
Suppl. Fig.10B
